# Reduced FEV_1_ as Prognostic Factors in Patients With Advanced NSCLC Receiving Immune Checkpoint Inhibitors

**DOI:** 10.3389/fmed.2022.860733

**Published:** 2022-03-22

**Authors:** Yi-Luen Shen, Chia-I Shen, Chi-Lu Chiang, Hsu-Ching Huang, Kun-Ta Chou, Chao-Hua Chiu, Yuh-Min Chen, Yung-Hung Luo

**Affiliations:** ^1^Department of Chest Medicine, Taipei Veterans General Hospital, Taipei, Taiwan; ^2^Division of Chest Medicine, Department of Internal Medicine, Asia University Hospital, Taichung, Taiwan; ^3^School of Medicine, National Yang Ming Chiao Tung University, Taipei, Taiwan; ^4^Institute of Clinical Medicine, National Yang Ming Chiao Tung University, Taipei, Taiwan

**Keywords:** forced expiratory volume (FEV) 1 second, immune checkpoint inhibitor (ICI), advanced non-small cell lung cancer, pulmonary function test (PFT), chronic lung disease (CLD)

## Abstract

**Background:**

The aim of study is to investigate the influence of pulmonary function on the prognosis in patients with advanced non-small cell lung cancer (NSCLC) receiving immune checkpoint inhibitors (ICI).

**Patients and Methods:**

Data were collected retrospectively from 151 patients with stage IV NSCLC who received ICI and completed spirometry before ICI therapy in Taipei Veterans General Hospital between January 2016 and December 2020. The co-primary end points were overall survival (OS) and progression-free survival (PFS) between groups divided by 80% predicted FEV_1_ since ICI therapy started; the secondary outcomes were objective response rate.

**Results:**

Among 151 patients enrolled to this study, 67.5% of patients were men, 75.5% were adenocarcinoma, 24.5% had known targetable driver mutation, 33.8% received first-line ICI, and 62.8% received ICI monotherapy. The objective response rate was 24.5% and disease control rate was 54.3%. In multivariable analysis, patient with reduced FEV_1_ had inferior PFS (FEV_1_ < 80% vs. FEV_1_ ≥ 80%, adjusted HR = 1.80, *P* = 0.006) and OS (FEV_1_ < 80% vs. FEV_1_ ≥ 80%, adjusted HR = 2.50, *P* < 0.001). Median PFS and OS in the preserved FEV_1_ group (≥80% predicted FEV_1_) compared to the reduced FEV_1_ group (<80% predicted FEV_1_) were 5.4 vs. 2.9 months (HR = 1.76, *P* = 0.003) and 34.9 vs. 11.1 months (HR = 2.44, *P* < 0.001), respectively. The other independent prognostic factors of OS include stage IVA disease (adjusted HR = 0.57, *P* = 0.037), initial liver metastasis (adjusted HR = 2.00, *P* = 0.049), ICI monotherapy (adjusted HR = 1.73, *P* = 0.042) and ICI related pneumonitis (adjusted HR = 3 .44, *P* = 0.025).

**Conclusions:**

Reduced FEV_1_ is strongly associated with inferior clinical outcomes in patients with advanced NSCLC treated with ICI.

## Introduction

Lung cancer is the leading cause of death worldwide, most of which is non-small cell lung cancer (NSCLC), accounting for 85% of all cases (1). In recent decades, several oncogenic molecular alterations such as *epidermal growth factor receptor* (*EGFR*) mutation, *anaplastic lymphoma kinase* (*ALK*) gene rearrangement, have been found and established as the targets of therapy. The management of NSCLC with targetable oncogene is remarkably advanced in past decade (2). However, the outcome of advanced NSCLC without targeted therapy remained dismal. Immune checkpoint inhibitors (ICI) represent another breakthrough achievement in cancer treatment. ICI which targets programmed cell death protein-1 (PD-1) or programmed cell death ligand-1 (PD-L1), have shown improvement of progression free survival (PFS) and overall survival (OS) in clinical trials, compared to chemotherapy in first-line or second-line therapy (3–7).

Lung cancer frequently developed in the patients with chronic lung disease, such as chronic obstructive pulmonary disease (COPD) or interstitial lung disease (ILD) (8–11). Chronic inflammation is the keystone pathogenesis of airway remodeling, mucus plugging, and parenchymal destruction (12). The negative impact of coexisting chronic lung disease on prognosis in lung cancer is well-known in previous studies (13–17). Furthermore, reduced forced expiratory volume in 1 second (FEV_1_) is recognized as poor prognostic factor in lung cancer (18–20).

Interestingly, recent studies on immunotherapy implicate that COPD is associated with better clinical outcomes in patients with NSCLC treated with ICI (21). Another immunological study suggests pre-existing ILD doesn't impact prognosis in patients treated with first-line pembrolizumab (22). To the best of our knowledge, the influence of reduced pulmonary function in patients with NSCLC treated with ICI is not fully investigated. The aim of study is to investigate the impact of pulmonary function on the prognosis and treatment outcome in patients treated with ICI.

## Methods

### Study Population

**Figure 1:**
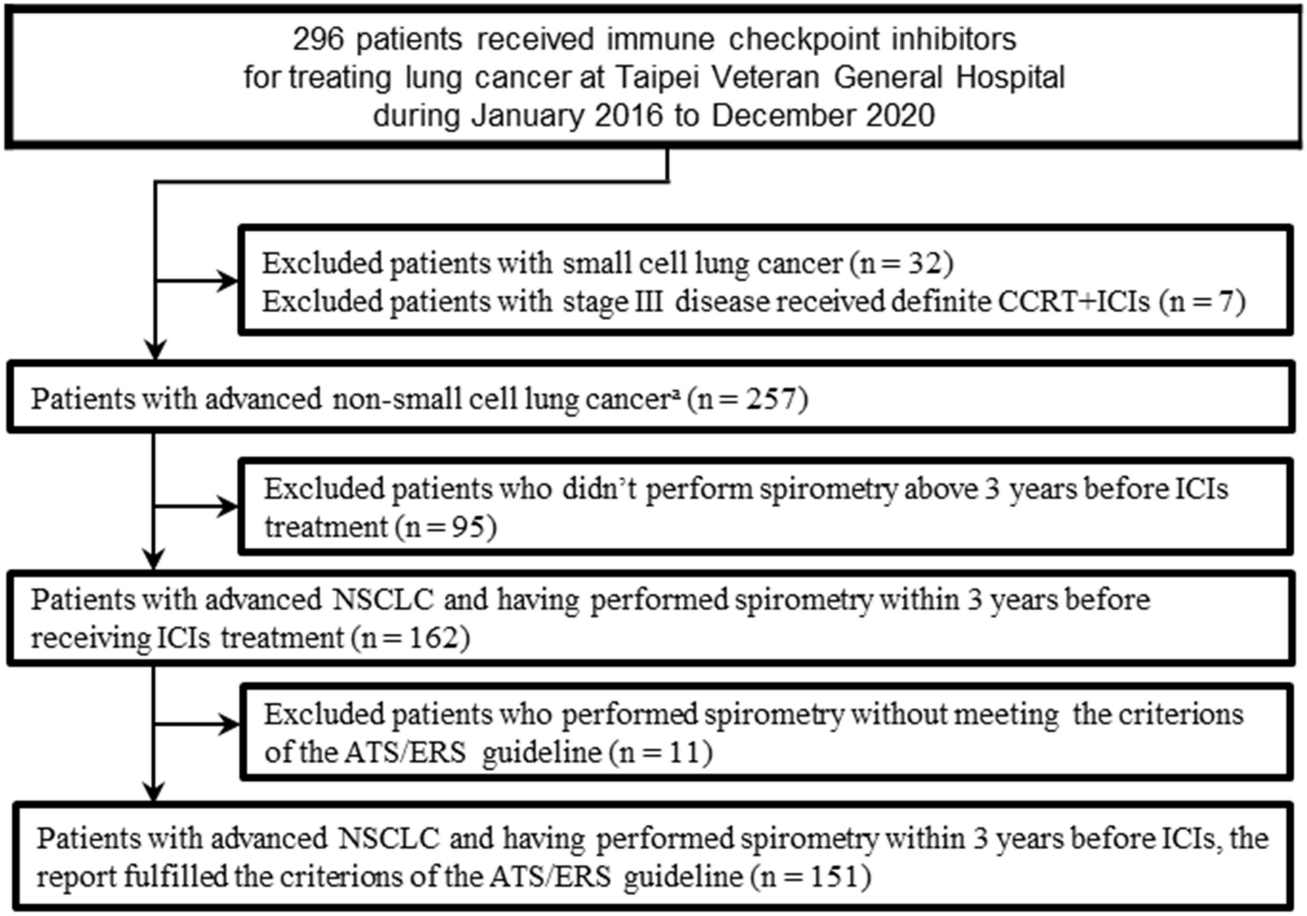
Flow chart of the study population. ATS, American Thoracic Society; CCRT, concurrent chemoradiotherapy; ERS, European Respiratory Society; ICI, immune checkpoint inhibitors; NSCLC, non-small cell lung cancer. ^a^Included adenocarcinomas, squamous cell carcinomas, adenosquamous carcinomas, large cell carcinomas, sarcomatoid carcinomas, large cell neuroendocrine carcinomas, and non–small-cell carcinomas not otherwise specified.

This is a retrospectively observational cohort study of patients with advanced NSCLC who received ICI. We identified 296 patients who received ICI between January 2016 and December 2020 from the lung cancer registry in Taipei Veterans General Hospital. Patients with small cell lung cancer (*N* = 32), or with stage III disease received definite concurrent chemoradiotherapy then followed by ICI (*N* = 7) were excluded. Then patients who received ICI treatment without undergoing spirometry (*N* = 95) before ICI, or those who underwent spirometry without meeting the criterions of the American Thoracic Society/European Respiratory Society (*N* = 11) were excluded. The final study population included 151 patients (Figure 1). Our study was carried out in accordance with the principles of the Declaration of Helsinki. The Institutional Review Board of Taipei Veterans General Hospital had approved our study (VGHIRB no. 2020-07-046CC) and waived the requirement for informed consent.

### Assessments and Data Collection

Data regarding patients' demographics, including age, sex, smoking history, Eastern Cooperative Oncology Group performance status (ECOG PS) (23), and tumor characteristics, such as stage, histology, initial metastatic sites, status of *EGFR* mutation or *ALK, c-ROS oncogene 1* (*ROS-1*) rearrangement, prior treatments and ICI-related pneumonitis (ICI-pneumonitis) were collected from electronic medical records. PD-L1 expression was assessed in formalin-fixed, paraffin-embedded tumor samples using PD-L1 IHC 22C3 pharmDx Kit (clone 22C3 [DAKO, Carpinteria, CA]), performed on Dako Autostainer Link 48 platform with a validated and automated staining protocol. The tumor proportion score (TPS) was defined as the percentage of viable tumor cells showing partial or complete membrane staining. Spirometry was performed using Vmax 22 (SensorMedics, Yorba Linda, CA), and interpretated according to the recommendations of the American Thoracic Society/European Respiratory Society guidelines (24, 25). The FEV_1_ (% of predicted), FVC (% of predicted), and FEV_1_/FVC (ratio) from pulmonary function test results were based on pre-bronchodilation values measured before ICI treatment. FEV_1_/FVC < 0.7 is classified as airflow obstruction, and 80% of predicted FEV_1_ was determined as a universal cut-off value for distinction between the reduced FEV_1_ group and preserved FEV_1_ group, according to previous studies and the classification of Global Initiative for Chronic Obstructive Lung Disease (GOLD) guideline (20, 26–28).

### Endpoints

The co-primary endpoints of this study were OS, measured from the date of starting ICI to death from any cause or last known date alive, and PFS, measured from the date of starting ICI to the date of initial disease progression, death from any cause, or the last date known to be alive without disease progression. The treatment response and the date of disease progression were confirmed by two authors (Y.L.S. and Y.H.L.) who reviewed the diagnostic imaging and medical record. The secondary endpoint was the objective response rate (ORR), defined as the percentage of patients with a confirmed complete response (CR) or partial response (PR). Treatment response was routinely reviewed every 2–3 months, or when disease progression was highly suspected. The assessment of response was based on Response Evaluation Criteria in Solid Tumors (RECIST, version 1.1) (29).

### Statistical Analysis

Categorical data from patients' profile were presented as numbers (%), and compared using Pearson's Chi-square-test and Fisher's exact test, as appropriate. Continuous variables were presented as means with standard deviation or median with interquartile range based on Kolmogorov-Smirnov normality test, then performed Student's *t*-test or Mann–Whitney *U*-test, respectively. The Kaplan–Meier method with the log-rank test was used for survival analysis. Hazard ratios (HRs) and 95% CI were calculated using the Cox proportional-hazard model, and multivariable analysis for baseline characteristics of patients and spirometry parameters. Multiple Cox proportional-hazard models were performed using the significant variables (*p* < 0.10) in the multivariate analysis. All tests were two-sided, and *p* < 0.05 were considered significant. All analyses were performed using SPSS software (version 25.0, IBM corp., Chicago, IL, USA).

## Results

### Patient Characteristics and Treatment

Among 151 patients receiving ICI, the mean age was 63.0 years old while started treatment; 102 (67.5%) were men and 83 patients had smoking history (55.0%). The majority of ECOG PS was 1-2 (90 patients, 59.6%), and other patients was 0 (61 patients, 40.4%). There were 58 patients at stage IVA (38.4%) and 93 patients at stage IVB or IVC (61.6%) before ICI, and the proportions of patients with initial distant metastasis of brain, lung, and liver were 22.5, 33.1, and 11.3%, respectively. Most of the histopathological type was adenocarcinoma (114 patients, 75.5%), then followed by squamous cell carcinoma (25 patients, 16.6%). Thirty-three patients presented *EGFR* mutation (21.9%); 1 patient had *ALK* rearrangement; 3 patients possessed *ROS-1* rearrangement, and 99 patients with EGFR wild type were documented without known driver mutation. The proportions of patients with PD-L1 TPS ≥ 50%, 1–49%, and <1% were 23.2, 17.9, and 19.9%, respectively. However, PD-L1 expression was unavailable in many patients (39.1%). Total 51 patients received first-line ICI therapy (33.8%). Previous-treated patient received ICI had all received chemotherapy before (100 patients), then 89 patients had previously received radiotherapy and 42 patients had lung surgery. There were 96 patients received ICI monotherapy (62.8%) and 55 patients received ICI treatment combined with other therapies, including anti-angiogenesis agent, chemotherapy, or others (37.2%). Total 6 (4%) patients had ICI-pneumonitis. For spirometry data, median FEV_1_ was 1.99 liters (84.0% median prediction of FEV_1_), and the median FVC was 2.65 liters (83.0% median prediction of FVC). The median FEV_1_/FVC ratio was 77.0% (Table 1). There was significant correlation between percentage of prediction of FEV_1_ and FVC in our study (*R* = 0.910, *P* < 0.001). Thirty-three patients had FEV_1_/FVC ratio <0.7, but only 7 patients had physician-diagnosed COPD in medical record. There are 65 patients with reduced FEV_1_. But only 22 patients had FEV_1_/FVC <0.7. Thirty-five patients were reported restrictive lung disease, about 11 of 35 patients had received lung surgery, 21 of 35 patients experienced radiotherapy. For other 8 patients were reported normal ventilatory function with preserved TLC, 6 of 8 patients had received radiotherapy and 1 patient was treated as asthma and received inhaled corticosteroid. There was 1 patient cannot be refined detailly based on electrical medical record.

**Table 1:** Demographics and characteristics of patients undergoing immune checkpoint inhibitors treatment and characteristics based on treatment response.

**Characteristics**	**Total (*N =* 151)**	**Treatment response**		
		**PD/SD (*N =*114)**	Responder^*^(*N =* 37)	***P-*value**
Age at ICI treatment (yrs)	63.0 ± 11.1	62.9 ± 10.6	63.2 ± 12.7	0.886
< 70 yrs	108 (71.5)	82 (71.9)	26 (70.3)	0.846
≥ 70 yrs	43 (28.5)	32 (28.1)	11 (29.7)	
Male sex	102 (67.5)	78 (68.4)	24 (64.9)	0.688
Smoking	83 (55.0)	61 (53.5)	22 (59.5)	0.527
ECOG				0.020
0	61 (40.4)	40 (35.1)	21 (55.8)	
1-2	90 (59.6)	74 (64.9)	16 (43.2)	
Stage at ICI treatment				0.101
IVA	58 (38.4)	48 (42.1)	10 (27.0)	
IVB & IVC	93 (61.6)	66 (57.9)	27 (73.0)	
**Initial distant metastasis**
Brain	34 (22.5)	29 (25.4)	5 (13.5)	0.131
Lung	50 (33.1)	41 (36.0)	9 (24.3)	0.191
Liver	17 (11.3)	12 (10.5)	5 (13.5)	0.565^†^
Pathology				0.848
Adenocarcinoma	114 (75.5)	85 (74.6)	29 (78.4)	
Squamous cell carcinoma	25 (16.6)	20 (17.5)	5 (13.5)	
Others	12 (7.9)	9 (7.9)	3 (8.1)	
Driver mutation				0.322^†^
EGFR WT	99 (65.6)	70 (61.4)	29 (78.4)	
EGFR Mu (+)	33 (21.9)	27 (23.7)	6 (16.2)	
Other mutation	4 (2.6)	4 (3.5)	0 (0.0)	
N/A	15 (9.9)	13 (11.4)	2 (5.4)	
PD-L1 expression				0.180
TPS < 1%	30 (19.9)	23 (20.2)	7 (18.9)	
1% ≤ TPS ≤ 49%	27 (17.9)	20 (17.5)	7 (18.9)	
TPS ≥ 50%	35 (23.2)	22 (19.3)	13 (35.1)	
N/A	59 (39.1)	49 (43.0)	10 (27.0)	
First-line therapy	51 (33.8)	34 (29.8)	17 (45.9)	0.072
≥ 2-line therapy	100 (66.2)	80 (70.2)	20 (54.1)	0.072
Prior C/T^**^	100 (66.2)	80 (70.2)	20 (54.1)	0.072
Prior R/T	89 (59.8)	71 (62.3)	18 (48.6)	0.143
Prior Surgery	42 (27.8)	34 (29.8)	8 (21.6)	0.333
ICI regimen				0.075
ICI monotherapy	96 (62.8)	77 (67.5)	19 (51.4)	
ICI combination therapy	55 (37.2)	37 (32.5)	18 (48.6)	
ICI-pneumonitis (all grade)	6 (4.0)	6 (5.3)	0 (0)	0.337^†^
FEV_1_ (L)	1.99 [1.41, 2.59]	1.97 [1.33, 2.59]	2.06 [1.45, 2.75]	0.314^‡^
FEV_1_ % pred (%)	84.0 [63.0, 99.0]	83.0 [62.0, 99.3]	86.0 [67.5, 100.0]	0.374^‡^
FVC (L)	2.65 [1.92, 3.42]	2.62 [1.90, 3.33]	2.94 [1.96, 3.53]	0.240^‡^
FVC % pred (%)	83.0 [67.0, 99.0]	82.5 [62.0, 98.0]	85.0 [74.5–100.5]	0.300^‡^
FEV_1_/FVC ratio (%)	77.0 [71.0, 82.0]	77.5 [70.0, 83.0]	76.0 [71.0, 81.0]	0.526^‡^

*Categorical variables are presented as frequency (percentage) and compared PD/SD and Responder with Pearson's Chi-square-test and Fisher's exact test. Continuous variables are performed Kolmogorov-Smirnov normality test initially. Age at ICI treatment pass normality test and it's presented as mean ± standard deviation and compared with Student's T-test. Pulmonary function test is record as median [interquartile range] and use non-parametric test with Mann-Whitney U-Test*.

*≥ 2-line, second-line or more therapy after previous treatment failure on advanced lung cancer; C/T, chemotherapy; ECOG, Eastern Cooperative Oncology Group performance status; EGFR Mu(+), EGFR mutation; EGFR WT, wild type of epidermal growth factor receptor; FEV_1_, forced expiratory volume in 1 second; FEV_1_% pred, percentage of predicted FEV_1_ FVC, forced vital capacity; FVC % pred, percentage of predicted FVC; ICI, immune checkpoint inhibitors; ICI combination therapy, immune checkpoint inhibitors combined with other anticancer therapy, included chemotherapy, anti-angiogenesis, or tyrosine kinase inhibitor; ICI-pneumonitis, immune checkpoint inhibitors related pneumonitis; N/A, not available; PD, disease progression based on RECIST 1.1; SD, stable disease based on RECIST 1.1; PD-L1, programmed death-receptor ligand-1; R/T, radiotherapy; TPS, tumor proportion score*.

* *Responder: only partial remission (PR) in this cohort study*.

** *All patients who initiated ICI as ≥ 2-line therapy had received chemotherapy before*.

† *Fisher's Exact Test*.

‡ *Mann-Whitney U-Test*.

### Treatment Response in Patients Receiving ICI

In first period of follow-up, the ORR based on RECIST was 24.5%, and the disease control rate was 54.3%. The demographics distribution of patients, categorized by treatment response, was not significantly different in levels of PD-L1 expression and other variables, except the ECOG PS. The proportion of ECOG PS = 0 in the group with treatment responder (with CR or PR) was higher than that in the non-responder (with stable disease or progressive disease) (*P* = 0.020) (Table 1). The detailed subgroup treatment response was shown in Supplementary Table 1. Among 19 variables, ECOG PS was an independent factor for prediction of treatment response in multivariable analysis (EGOG PS = 1-2 vs. ECOG PS = 0, adjusted odds ratio = 0.42, *P* = 0.026) (Supplementary Table 2).

### Progression Free Survival in Patients Treated With ICI

**Figure 2:**
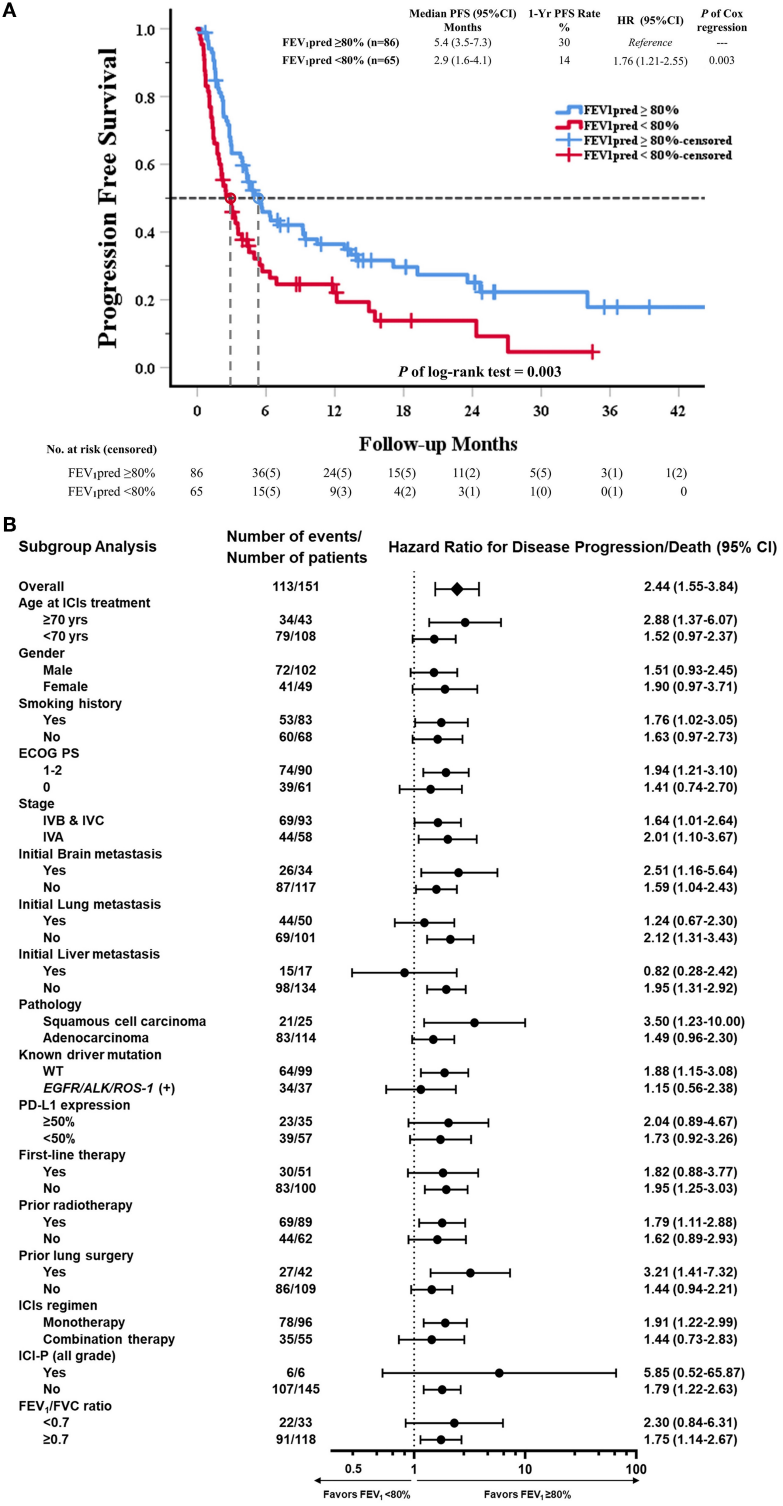
Kaplan–Meier curve of PFS and subgroup analysis of PFS in all patients received ICI (80% of predicted FEV_1_ as a cut-off value of FEV_1_). **(A)** The Kaplan–Meier curve estimates of PFS, according to using 80% of predicted FEV_1_ as a cut-off value. Tick marks represent data censored at the last time the patient was known to be alive and without disease progression. Progression free survival was assessed according to Response Evaluation Criteria in Solid Tumors (RECIST), version 1.1, and the data reviewed by authors group. **(B)** The data sheet and Forest plot shows the analysis of PFS in all subgroups. Vertical dotted line in subgroup analysis represents HR, showing PFS benefit for FEV_1_pred ≥80% compared with FEV_1_pred < 80% in all subgroups evaluated.

In Kaplan-Meier survival analysis, median PFS in reduced FEV_1_ group (FEV_1_ pred <80%) was significantly shorter than in preserved FEV_1_ group (FEV_1_ pred≥80%) (Median PFS: 2.9 vs. 5.6 months; HR = 1.76, *P* = 0.003) (Figure 2A). The subgroup analysis of PFS showed that an increased risk of disease progression/death with reduced FEV_1_ was found in most subgroups (Figure 2B).

In univariable Cox regression models of PFS, known prognostic factors including sex, smoking, ECOG PS, lung/liver metastasis, first-line therapy, prior radiotherapy, ICI regimen, ICI-pneumonitis, and reduced FEV_1_ were significant variables, which were then analyzed in multivariable Cox models (Supplementary Table 3). In multivariable analysis, reduced FEV_1_ group showed higher risk for disease progression compared to preserved FEV_1_ group (adjusted HR = 1.80, *P* = 0.006), and other 5 independent factors were: ECOG PS = 1-2 (adjusted HR = 2.08, *P* < 0.001), initial lung metastasis (adjusted HR = 1.69, *P* = 0.014), initial liver metastasis (adjusted HR = 2.36, *P* = 0.005), prior radiotherapy (adjusted HR = 1.72, *P* = 0.009), ICI-pneumonitis (adjusted HR = 3.78, *P* = 0.003) (Table 2).

**Table 2:** Factors associated with progression free survival by Cox regression model in all patients received immune checkpoint inhibitors (80% of predicted FEV_1_ as a cut-off value of FEV_1_).

**Variable**	***N***	**Multivariable analysis** (***P*** **<** **0.1)**	
		**HR (95% CI)**	***P-*value**
Gender			0.111
Female	49	*Reference*	
Male	102	0.67 (0.41–1.10)	
Smoking history			0.837
No	68	*Reference*	
Yes	83	0.95 (0.60–1.52)	
ECOG PS			0.001
0	61	*Reference*	
1-2	90	2.08 (1.35–3.20)	
Initial lung metastasis			0.014
No	101	*Reference*	
Yes	50	1.69 (1.11–2.56)	
Initial liver metastasis			0.005
No	134	*Reference*	
Yes	17	2.36 (1.29–4.29)	
First-line therapy			0.180
No	100	*Reference*	
Yes	51	0.69 (0.41–1.18)	
Prior radiotherapy			0.009
No	62	*Reference*	
Yes	89	1.72 (1.15–2.59)	
ICIs regimen			0.305
Monotherapy	96	1.27 (0.80–2.02)	
Combination therapy	55	*Reference*	
ICI-pneumonitis (all grade)			0.003
No	145	*Reference*	
Yes	6	3.78 (1.57–9.11)	
FEV_1_ pred(%)			0.006
Preserved FEV_1_ (≥ 80%)	86	*Reference*	
Reduced FEV_1_ (< 80%)	65	1.80 (1.18–2.74)	

*In total 119 events of progression or death in this study, we selected those variables which P <0.1 in univariate analysis, and perform multivariable Cox regression analysis for PFS (See detail full Cox regression analysis of PFS in Supplementary Table 2)*.

*Combination therapy, immune checkpoint inhibitors combined with other anticancer therapy, included chemotherapy, anti-angiogenesis, or tyrosine kinase inhibitor; ECOG PS, Eastern Cooperative Oncology Group performance status; EGFR, epidermal growth factor receptor mutation; FEV_1_ pred(%), percentage of predicted FEV_1_; HR, hazard ratio; ICI, immune checkpoint inhibitors; ICI-pneumonitis, immune checkpoint inhibitor related pneumonitis*.

### Overall Survival in Patients Treated With ICI

**Figure 3:**
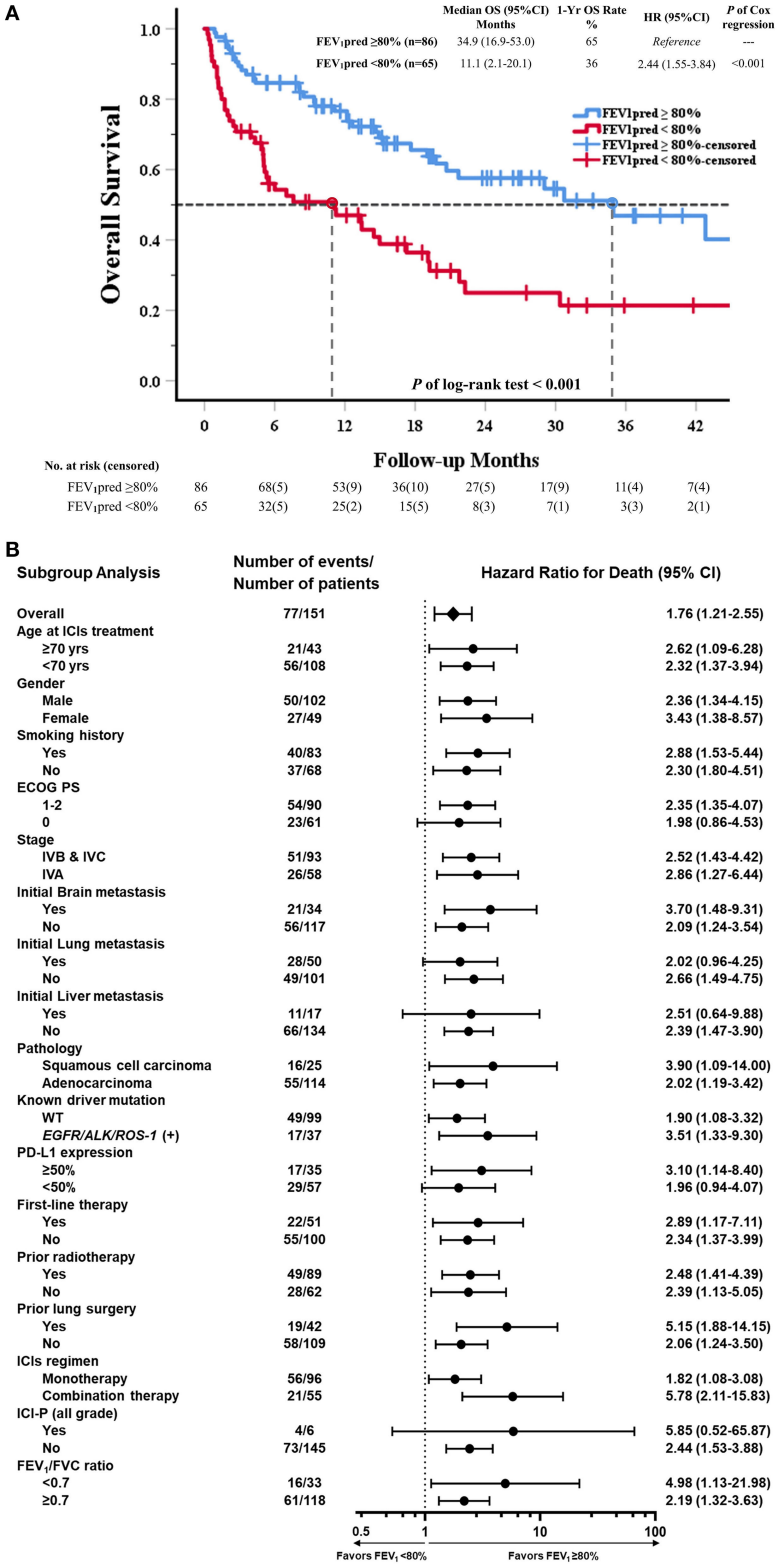
Kaplan–Meier curve of OS and subgroup analysis of OS in all patients received ICI (80% of predicted FEV_1_ as a cut-off value of FEV_1_). **(A)** The Kaplan–Meier curve estimates of OS, according to using 80% of predicted FEV_1_ as a cut-off value. Tick marks represent data censored at the last time the patient was known to be alive. **(B)** The data sheet and Forest plot shows the analysis of OS in all subgroups. Vertical dotted line in subgroup analysis represents HR, showing OS benefit for FEV_1_pred ≥80% compared with FEV_1_pred <80% in all subgroups evaluated.

Total 82 events of death were record in study cohort. In Kaplan-Meier survival analysis, OS in reduced FEV_1_ group was significantly shorter than in preserved FEV_1_ group. (Median OS: 11.1 vs. 34.9 months; HR = 2.44, *p* < 0.001) (Figure 3A). The subgroup analysis of OS showed that an increased risk of death with reduced FEV_1_ was found in most subgroups (Figure 3B).

In univariable Cox regression models of OS, known prognostic factors including ECOG PS, stage, brain/liver metastasis, prior radiotherapy, ICIs regimen, ICI-pneumonitis, and reduced FEV_1_ were significant variables, which were then analyzed in multivariable Cox models (Supplementary Table 4). In multivariable analysis, reduced FEV_1_ group presented higher risk of mortality than preserved FEV_1_ group (adjusted HR = 2.50, *P* < 0.001). Other 5 independent factors, including ECOG PS (adjusted HR = 1.90, *P* = 0.013), stage IVA disease (adjusted HR = 0.57, *P* = 0.037), initial liver metastasis (adjusted HR = 2.00, *P* = 0.049), and ICI-pneumonitis (adjusted HR = 3.44, *P* = 0.025) (Table 3).

**Table 3:** Factors associated with overall survival by Cox regression model in all patients received immune checkpoint inhibitors (80% of predicted FEV_1_ as a cut-off value of FEV_1_).

**Variable**	***N***	**Multivariable analysis** (***P*** **<** **0.1)**	
		**HR (95% CI)**	***P-*value**
ECOG PS			0.013
0	61	*Reference*	
1-2	90	1.90 (1.14–3.16)	
Stage			0.037
IVA	58	0.57 (0.34–0.97)	
IVB & IVC	93	*Reference*	
Initial brain metastasis			0.701
No	117	*Reference*	
Yes	34	1.11 (0.65–1.91)	
Initial liver metastasis			0.049
No	134	*Reference*	
Yes	17	2.00 (1.00–4.00)	
Prior radiotherapy			0.112
No	62	*Reference*	
Yes	89	1.50 (0.91–2.47)	
ICIs regimen			0.060
Monotherapy	96	1.65 (0.98–2.77)	
Combination therapy	55	*Reference*	
ICI-pneumonitis (all grade)			0.025
No	145	*Reference*	
Yes	6	3.44 (1.17–10.09)	
FEV_1_ pred (%)			<0.001
Preserved FEV_1_ (≥80%)	86	*Reference*	
Reduced FEV_1_ (<80%)	65	2.50 (1.56–3.99)	

*Total 82 events of death were record in study cohort, we selected those variables which P < 0.1 in univariate analysis, and perform multivariable Cox regression analysis for OS (See detail full Cox regression analysis of OS in Supplementary Table 3)*.

*Combination therapy, immune checkpoint inhibitors combined with other anticancer therapy, included chemotherapy, anti-angiogenesis, or tyrosine kinase inhibitor; ECOG PS, Eastern Cooperative Oncology Group performance status; EGFR, epidermal growth factor receptor mutation; FEV_1_ pred(%), percentage of predicted FEV_1_; HR, hazard ratio; ICI, immune checkpoint inhibitors; ICI-pneumonitis, immune checkpoint inhibitor related pneumonitis*.

Reduced FEV_1_ group also presented increased risk of disease progression or death in analysis of key subgroups (Different lines of therapy, patients without driver mutation; detail in Supplementary Data).

## Discussion

To date, this is the first study to analyze the treatment outcome of stage IV NSCLC receiving ICI in terms of lung function. Our study demonstrated that reduced FEV_1_, not reduced FEV_1_/FVC ratio, is an independent prognostic factor of the inferior survival outcome in patients with ICI-treated advanced NSCLC, irrespective of ECOG PS, various degrees of distant metastasis, different lines of therapy, ICI-combination therapy and ICI-pneumonitis.

In clinical practice, FEV_1_ is a non-invasive tool for evaluating pulmonary function. Patient who has declined FEV_1_ might be associated with increased respiratory symptoms, inferior life quality, and mortality (30–32). Past studies showed FEV_1_ worked as a better predictor than FVC in survival (33). A previous research discloses that patients with small cell lung cancer and FEV_1_ < 80% had inferior OS compared to those with FEV_1_≥ 80% (27). According to the American Society of Clinical Oncology guideline, baseline spirometry is recommended for every patient with cancer prior to ICI (34). However, it is not routinely tested in real-world experience. In most clinical settings, spirometry was performed for preoperative evaluation, pre-radiotherapy assessment, evaluating the cause of persisted dyspnea, or follow-up for chronic lung diseases, such as COPD or ILD.

Our study tested the hypothesis that using 80% predicted value of FEV_1_ in patients with advanced NSCLC receiving ICIs could meet the prognostic significance in OS and PFS. Previous two studies have reported that 50% predicted value of FEV_1_ at initial diagnosis of lung cancer is an independent prognostic factor for advanced NSCLC, after adjusting TNM stage and the presence of malignant pleural effusion, suggesting reduced FEV_1_ is an important factor in survival prediction (18, 19). Additionally, our study focuses on pre-treatment pulmonary functions, considering that initial pulmonary functions would be affected by subsequent treatment such as palliative surgical resection, radiotherapy, or drug-induced lung toxicities. Collectively, these findings suggest that patients with reduced FEV_1_ had inferior outcome, and their pulmonary function should be carefully monitored. Lung function forms part of exercise performance and quality of life. Assessment of respiratory symptoms and health related quality of life might be important to clarify the relationship between FEV_1_ and mortality.

In an earlier study, patients coexisting with COPD and NSCLC who received pembrolizumab monotherapy had longer PFS and OS than those without COPD (21). Nonetheless, our study did not show significant correlation between COPD and outcome. We used pre-bronchodilator spirometry in this study due to the limited number of patients with post-bronchodilator spirometry, which estimate the prevalence of COPD should be careful (35). Surprisingly, total 33 patients had FEV_1_/FVC <0.7 but only 7 patients had physician-diagnosed COPD in electrical medical records, which indicated COPD might be underestimated. Clinical physicians might overlook COPD or other chronic lung diseases and attribute respiratory symptoms to lung cancer in real-world practice (36). Inappropriate management of comorbidity might be fatal. The importance of pulmonary function tests for improving the clinical practice in comorbidity management of those patients with lung cancer is heightened by our findings in the current study.

In consideration of the ECOG PS, tumor stage, and ICI regimen, reduced FEV_1_ is still statistically significant with the risk of death, implicating that reduced FEV_1_ might interfere ICI treatment via some ambiguous mechanisms. Reduced FEV_1_ could be resulted from chronic lung disease in several pathways, such as recurrent infection, airway inflammation, mucus plugging, structural change of alveoli. Chronic engagement of checkpoint receptors with frequent inflammation and antigenic stimulation would lead to T cell exhaustion. Previous research disclosed that increasing PD-1 expression of CD8^+^ tumor-infiltrating T lymphocytes is found in patients coexisting with NSCLC and COPD, correlated to the level of reduced FEV_1_ (37). Given that T-cell exhaustion is the self-protective mechanism for dysregulation of immune reaction, patients with impaired lung function might be vulnerable to ICI therapy as blockade of PD-1/PD-L1 pathway, causing airway injury, lung function decline, or pneumonitis. Additionally, analysis of circulating inflammatory markers related to ICI treatment such as neutrophil to lymphocyte ratio, LDH or CRP, which are widely used in previous studies, could provide more detailed inflammatory profiles for evaluation in the future investigation (38, 39).

Interestingly, in this study, ICI-pneumonitis was also associated with poorer PFS and OS. ICI-pneumonitis would impair pulmonary function seriously, and lead to mortality and morbidity if left untreated. However, our study didn't investigate all types of immune-related adverse events (irAE), which might be related to variable treatment response (40, 41). Consequently, reduced FEV_1_ before treatment may be potentially exacerbated by ICIs, resulting in deterioration of pulmonary function and poor survival outcome.

Based on this study, pretreatment pulmonary function is a potentially immunotherapeutic parameter that should be regularly performed before ICI administration, regardless of previous treatment status. It also provides more information for clinicians regarding comprehensive evaluation of illness. In real-world practice, the prognosis for previous-treated patients remains dismal, the useful parameters are urgently needed clinically for these patients. Further prospective studies for longitudinal investigation of the dynamic changes of pulmonary function before and after ICI are warranted, and could help us elucidate the relationship between pulmonary function and prognosis of patients receiving ICIs.

There are several limitations of in our study. First, this is a retrospective cohort study in a single tertiary medical center, and some relevant data including all types of irAE, cumulative dose of ICI, subsequent treatment after ICI failure, tumor infiltrated lymphocytes, and tumor mutational burden were not available. Besides, there were about 39% of all patients misses the PD-L1 study owing to lack of adequate biopsy tissue or not performed. Those missing data may influence the statistical analysis. Second, our study population was relatively small and had limited generalizability due to a single center experience and lack of validation cohort. Third, we used spirometry test as closely as possible before ICI treatment, but it might remain unsatisfied for the perfect timing of test. Additionally, the follow-up spirometry for lung function decline was unavailable for comprehensive study. Fourth, lung volume test, bronchodilator test and diffusing capacity are not routinely performed in most patients, so the impact of restrictive lung disease or air trapping on the outcome is not fully investigated. Despite the limitations, our data are still representative in the real-world practice and useful for patients who undergo ICI therapy for NSCLC.

In conclusion, reduced FEV_1_ with cut-off level of 80% predicted value is strongly associated with inferior outcomes in patients with advanced NSCLC treated with ICIs. Regular follow-up of spirometry might facilitate more accurate prediction of prognosis, thereby assisting in optimal decision-making in patients with ICI treatment. The detailed pathophysiology regarding the influences of reduced FEV_1_ on patient's prognosis remains to be elucidated, and further perspective study is warranted.

## Data Availability Statement

The original contributions presented in the study are included in the article/Supplementary Material, further inquiries can be directed to the corresponding author.

## Ethics Statement

The studies involving human participants were reviewed and approved by the Institutional Review Board of Taipei Veterans General Hospital (VGHIRB no. 2020-07-046CC). Written informed consent for participation was not required for this study in accordance with the national legislation and the institutional requirements.

## Author Contributions

Y-LS: conceptualization, methodology, software, formal analysis, writing—original draft, and visualization. C-IS: conceptualization, methodology, formal analysis, investigation, resources, and data curation. C-LC, H-CH, and K-TC: investigation, resources, and data curation. C-HC: investigation, resources, data curation, and funding acquisition. Y-MC: investigation resources, supervision, project administration, and funding acquisition. Y-HL: conceptualization, formal analysis, investigation, resources, data curation, writing—review and editing, supervision, project administration, and funding acquisition. All authors contributed to the article and approved the submitted version.

## Funding

This research was funded by the Ministry of Science and Technology (MOST) (108-2628-B-075-007, 109-2628-B-07-023, 109-2628-B-075-023, 110-2314-B-075-078-MY3, and 110-2811-B-075-513), Taipei Veterans General Hospital (V108D46-004-MY2-2, V108E-006-43, V109C-123, V109E-007-3, V110C-140, V111C-138, and V111E-001-3), Yen-Tjing-Ling Medical Foundation (CI-111-10), Melissa Lee Cancer Foundation (MLCF-V111_A11105), and the Department of Health Cancer Center Research of Excellence (MOHW109-TDU-B-211-134019 and MOHW110-TDU-B-211-144019), Taiwan.

## Conflict of Interest

C-HC has received honoraria from AstraZeneca, Boehringer-Ingelheim, Bristol-Myers Squibb, Chugai Pharmaceutical, Eli Lilly, Merck Sharp & Dohme, Novartis, Ono Pharmaceutical, Pfizer, Roche, and Takeda. C-LC has received speaking honoraria from AstraZeneca, Boehringer Ingelheim, Bristol-Myers Squibb, Chugai Pharmaceutical, Merck Sharp & Dohme, Novartis, Pfizer, and Roche. C-IS has received support for attending meetings from Merck Sharp & Dohme. The remaining authors declare that the research was conducted in the absence of any commercial or financial relationships that could be construed as a potential conflict of interest.

## Publisher's Note

All claims expressed in this article are solely those of the authors and do not necessarily represent those of their affiliated organizations, or those of the publisher, the editors and the reviewers. Any product that may be evaluated in this article, or claim that may be made by its manufacturer, is not guaranteed or endorsed by the publisher.
