# Supplementary material for: Reduced FEV1 as Prognostic Factors in Patients With Advanced NSCLC Receiving Immune Checkpoint Inhibitors
Source: Front Med (Lausanne). 2022 Mar 22;9:860733. doi: 10.3389/fmed.2022.860733 (PMC8980716; doi:10.3389/fmed.2022.860733)
Supplement: Supplementary file 1 [file Data_Sheet_1.DOCX]

**Appendix**

**Supplementary Table 1: Treatment response in first period of follow-up**

**— p.1**

**Supplementary Table 2: Factors associated with treatment responder of all patients who received immune checkpoint inhibitor (ICI)**

**— p.2**

**Supplementary Table 3: Cox regression model of progression free survival (PFS) in all patients received ICI (80% of predicted FEV_1_ as a cut-off value of FEV_1_)**

**— p.4**

**Supplementary Table 4: Cox regression model of overall survival (OS) in all patients received ICI (80% of predicted FEV_1_ as a cut-off value of FEV_1_)**

**— p.6**

**Supplementary Figure 1-2 & Table 5-6: Kaplan–Meier curve and Cox regression model of PFS and OS, in all patients received ICI (4 groups of FEV_1_ as a cut-off value of FEV_1_)**

**— p.8**

**Supplement Figure 3-4 & Table 7-8: Subgroup analysis for first-line ICI─**

**Kaplan–Meier curve and Cox regression model of PFS and OS**

**— p.14**

**Supplementary Figure 5-6 & Table 9-10: Subgroup analysis for ≥second-line ICI─**

**Kaplan–Meier curve and Cox regression model of PFS and OS**

**— p.20**

**Supplementary Figure 7-8 & Table 11-12: Subgroup analysis for patient without driver mutation─Kaplan–Meier curve and Cox regression model of PFS and OS**

**— p.26**

**Supplementary Table 1: Treatment response in first period of follow-up**

| **Variable** | **Total** | **Treatment Response** | | | | | | |
| --- | --- | --- | --- | --- | --- | --- | --- | --- |
|  |  | **CR** | **PR** | **SD** | **PD** | **N/A** | **ORR(%)** | **DCR(%)** |
| **Overall** | **151** | **0** | **37** | **45** | **68** | **1** | **24.5** | **54.3** |
| **ICI Treatment** |  | **CR** | **PR** | **SD** | **PD** | **N/A** | **ORR(%)** | **DCR(%)** |
| **First-line therapy** |  |  |  |  |  |  |  |  |
| **ICI monotherapy** | **20** | **0** | **5** | **8** | **7** | **0** | **20.0** | **65.0** |
| **ICI combination** | **31** | **0** | **12** | **9** | **10** | **0** | **38.7** | **67.7** |
| **≥ Second-line therapy** |  |  |  |  |  |  |  |  |
| **ICI monotherapy** | **76** | **0** | **14** | **24** | **37** | **1** | **18.4** | **50.0** |
| **ICI combination** | **24** | **0** | **6** | **4** | **14** | **0** | **25.0** | **41.7** |
| **Tumor Characteristics** |  | **CR** | **PR** | **SD** | **PD** | **N/A** | **ORR(%)** | **DCR(%)** |
| **EGFR mutant** | **33** | **0** | **6** | **11** | **16** | **0** | **18.2** | **51.5** |
| **PD-L1 expression** |  |  |  |  |  |  |  |  |
| **<1%** | **30** | **0** | **7** | **8** | **14** | **1** | **23.3** | **50.0** |
| **1-49%** | **27** | **0** | **7** | **8** | **12** | **0** | **25.9** | **55.6** |
| **≥50%** | **35** | **0** | **13** | **8** | **14** | **0** | **37.1** | **60.0** |
| **Pulmonary function** |  | **CR** | **PR** | **SD** | **PD** | **N/A** | **ORR(%)** | **DCR(%)** |
| **FEV_1_/FVC ratio** |  |  |  |  |  |  |  |  |
| **≥0.7** | **118** | **0** | **29** | **35** | **54** | **0** | **24.6** | **54.2** |
| **<0.7** | **33** | **0** | **8** | **10** | **14** | **1** | **24.2** | **54.5** |
| **FEV_1_ pred(%)** |  |  |  |  |  |  |  |  |
| **≥ 80%** | **86** | **0** | **24** | **29** | **32** | **1** | **27.9** | **62.8** |
| **< 80%** | **65** | **0** | **13** | **16** | **36** | **0** | **20.0** | **44.6** |

Abbreviation: Combination therapy: immune checkpoint inhibitors combined with other anticancer therapy, included chemotherapy, anti-angiogenesis, or tyrosine kinase inhibitor; CR: complete response; DCR: disease control rate = (CR+PR+SD)/Total; *EGFR*: *epidermal growth factor receptor* mutation; FEV_1_: forced expiratory volume in 1 second; FEV_1_ pred(%): percentage of predicted FEV_1_; FVC: forced vital capacity; ICI: immune checkpoint inhibitors; ORR: overall response rate = (CR+PR)/ Total; N/A: cannot be evaluated/ not available; PD: progressive disease; PD-L1: programmed cell death ligand-1; PR: partial response; performance status; SD: stable disease.

**Supplementary Table 2. Factors associated with treatment responder of all patients who received ICI**

|  |  | **Univariate analysis** | |  | **Multivariable analysis (*P* <0.1)** | |  |
| --- | --- | --- | --- | --- | --- | --- | --- |
| **Variable** | **N** | **OR (95% CI)** | ***P* value** |  | **OR (95% CI)** | ***P* value** |  |
| Age at ICIs treatment |  |  | 0.846 |  |  |  |  |
| <70 yrs | 108 | *Reference* |  |  |  |  |  |
| ≥70 yrs | 43 | 1.08 (0.48-2.45) |  |  |  |  |  |
| Gender |  |  | 0.688 |  |  |  |  |
| Female | 49 | *Reference* |  |  |  |  |  |
| Male | 102 | 0.85 (0.39-1.86) |  |  |  |  |  |
| Smoking history |  |  | 0.528 |  |  |  |  |
| No | 68 | *Reference* |  |  |  |  |  |
| Yes | 83 | 1.27 (0.60-2.70) |  |  |  |  |  |
| ECOG PS |  |  | 0.021 |  |  | 0.026 |  |
| 0 | 61 | *Reference* |  |  | *Reference* |  |  |
| 1-2 | 90 | 0.41 (0.19-0.88) |  |  | 0.42 (0.19-0.90) |  |  |
| Stage |  |  | 0.105 |  |  |  |  |
| IVA | 58 | 0.51 (0.23-1.15) |  |  |  |  |  |
| IVB & IVC | 93 | *Reference* |  |  |  |  |  |
| Initial Brain metastasis |  |  | 0.138 |  |  |  |  |
| No | 117 | *Reference* |  |  |  |  |  |
| Yes | 34 | 0.46 (0.16-1.29) |  |  |  |  |  |
| Initial Lung metastasis |  |  | 0.194 |  |  |  |  |
| No | 101 | *Reference* |  |  |  |  |  |
| Yes | 50 | 0.57 (0.25-1.32) |  |  |  |  |  |
| Initial Liver metastasis |  |  | 0.618 |  |  |  |  |
| No | 134 | *Reference* |  |  |  |  |  |
| Yes | 17 | 1.33 (0.44-4.06) |  |  |  |  |  |
| Pathology |  |  | 0.568 |  |  |  |  |
| Squamous cell carcinoma | 25 | *Reference* |  |  |  |  |  |
| Adenocarcinoma | 114 | 1.37 (0.47-3.97) |  |  |  |  |  |
| Known driver mutation |  |  | 0.126 |  |  |  |  |
| WT | 99 | *Reference* |  |  |  |  |  |
| *EGFR/ALK/ROS-1* (+) | 37 | 0.47 (0.18-1.24) |  |  |  |  |  |
| PD-L1 expression |  |  | 0.201 |  |  |  |  |
| <50% | 57 | *Reference* |  |  |  |  |  |
| ≥50% | 35 | 1.82 (0.73-4.52) |  |  |  |  |  |
| First-line therapy |  |  | 0.074 |  |  | 0.192 |  |
| No | 100 | *Reference* |  |  | *Reference* |  |  |
| Yes | 51 | 2.00 (0.93-4.28) |  |  | 1.74 (0.76-4.00) |  |  |
| Prior radiotherapy |  |  | 0.145 |  |  |  |  |
| No | 62 | *Reference* |  |  |  |  |  |
| Yes | 89 | 0.57 (0.27-1.21) |  |  |  |  |  |
| Prior lung surgery |  |  | 0.336 |  |  |  |  |
| No | 109 | *Reference* |  |  |  |  |  |
| Yes | 42 | 0.65 (0.27-1.56) |  |  |  |  |  |
| ICI regimen |  |  | 0.078 |  |  | 0.293 |  |
| Monotherapy | 96 | 0.51 (0.24-1.08) |  |  | 0.64 (0.28-1.47) |  |  |
| Combination therapy | 55 | *Reference* |  |  | *Reference* |  |  |
| ICI-pneumonitis (all grade) |  |  | >0.999 |  |  |  |  |
| No | 145 | *Reference* |  |  |  |  |  |
| Yes | 6 | 0^*^ |  |  |  |  |  |
| FEV_1_/FVC ratio |  |  | 0.969 |  |  |  |  |
| ≥0.7 | 118 | *Reference* |  |  |  |  |  |
| <0.7 | 33 | 0.98 (0.40-2.42) |  |  |  |  |  |
| FEV_1_ pred(%) |  |  | 0.265 |  |  |  |  |
| Preserved FEV_1_ (≥ 80%) | 86 | *Reference* |  |  |  |  |  |
| Reduced FEV_1_ (< 80%) | 65 | 0.65 (0.30-1.39) |  |  |  |  |  |

Abbreviation: *ALK*: *anaplastic lymphoma kinase* gene rearrangement; Combination therapy: immune checkpoint inhibitors combined with other anticancer therapy, included chemotherapy, anti-angiogenesis, or tyrosine kinase inhibitor; ECOG PS: Eastern Cooperative Oncology Group performance status; *EGFR*: *epidermal growth factor receptor* mutation; FEV_1_: forced expiratory volume in 1 second; FEV_1_ pred(%): percentage of predicted FEV_1_; FVC: forced vital capacity; ICI: immune checkpoint inhibitors; ICI-pneumonitis: immune checkpoint inhibitor related pneumonitis; OR: odds ratio; PD-L1: programmed cell death ligand-1; PS: performance status; *ROS-1*: *cROS oncogene-1* rearrangement; WT: wild type of epidermal growth factor receptor.

^*^ No responder in ICI-pneumonitis (yes) group.

**Supplementary Table 3: Cox regression model of PFS in all patients received ICI (80% of predicted FEV_1_ as a cut-off value of FEV_1_)**

|  |  | **Univariate analysis** | |  | **Multivariable analysis(*P* <0.1)** | |  |
| --- | --- | --- | --- | --- | --- | --- | --- |
| **Variable** | **N** | **HR (95% CI)** | ***P v*alue** |  | **HR (95% CI)** | ***P* value** |  |
| Age at ICIs treatment |  |  | 0.276 |  |  |  |  |
| <70 yrs | 108 | *Reference* |  |  |  |  |  |
| ≥70 yrs | 43 | 1.25 (0.84-1.87) |  |  |  |  |  |
| Gender |  |  | 0.046 |  |  | 0.111 |  |
| Female | 49 | *Reference* |  |  | *Reference* |  |  |
| Male | 102 | 0.68 (0.46-0.99) |  |  | 0.67 (0.41-1.10) |  |  |
| Smoking history |  |  | 0.011 |  |  | 0.837 |  |
| No | 68 | *Reference* |  |  | *Reference* |  |  |
| Yes | 83 | 0.62 (0.43-0.90) |  |  | 0.95 (0.60-1.52) |  |  |
| ECOG PS |  |  | 0.003 |  |  | 0.001 |  |
| 0 | 61 | *Reference* |  |  | *Reference* |  |  |
| 1-2 | 90 | 1.83 (1.24-2.71) |  |  | 2.08 (1.35-3.20) |  |  |
| Stage |  |  | 0.450 |  |  |  |  |
| IVA | 58 | 0.86 (0.59-1.26) |  |  |  |  |  |
| IVB & IVC | 93 | *Reference* |  |  |  |  |  |
| Initial Brain metastasis |  |  | 0.164 |  |  |  |  |
| No | 117 | *Reference* |  |  |  |  |  |
| Yes | 34 | 1.38 (0.88-2.12) |  |  |  |  |  |
| Initial Lung metastasis |  |  | 0.048 |  |  | 0.014 |  |
| No | 101 | *Reference* |  |  | *Reference* |  |  |
| Yes | 50 | 1.38 (0.88-2.12) |  |  | 1.69 (1.11-2.56) |  |  |
| Initial Liver metastasis |  |  | 0.020 |  |  | 0.005 |  |
| No | 134 | *Reference* |  |  | *Reference* |  |  |
| Yes | 17 | 1.91 (1.10-3.29) |  |  | 2.36 (1.29-4.29) |  |  |
| Pathology |  |  | 0.547 |  |  |  |  |
| Squamous cell carcinoma | 25 | *Reference* |  |  |  |  |  |
| Adenocarcinoma | 114 | 0.86 (0.53-1.39) |  |  |  |  |  |
| Known driver mutation |  |  | 0.125 |  |  |  |  |
| WT | 99 | *Reference* |  |  |  |  |  |
| *EGFR/ALK/ROS-1* (+) | 37 | 1.39 (0.91-2.11) |  |  |  |  |  |
| PD-L1 expression |  |  | 0.826 |  |  |  |  |
| <50% | 57 | *Reference* |  |  |  |  |  |
| ≥50% | 35 | 1.06 (0.63-1.78) |  |  |  |  |  |
| First-line therapy |  |  | 0.011 |  |  | 0.180 |  |
| No | 100 | *Reference* |  |  | *Reference* |  |  |
| Yes | 51 | 0.58 (0.38-0.89) |  |  | 0.69 (0.41-1.18) |  |  |
| Prior radiotherapy |  |  | 0.068 |  |  | 0.009 |  |
| No | 62 | *Reference* |  |  | *Reference* |  |  |
| Yes | 89 | 1.43 (0.98-2.09) |  |  | 1.72 (1.15-2.59) |  |  |
| Prior lung surgery |  |  | 0.199 |  |  |  |  |
| No | 109 | *Reference* |  |  |  |  |  |
| Yes | 42 | 0.75 (0.49-1.16) |  |  |  |  |  |
| ICIs regimen |  |  | 0.007 |  |  | 0.305 |  |
| Monotherapy | 96 | 1.73 (1.16-2.58) |  |  | 1.27 (0.80-2.02) |  |  |
| Combination therapy | 55 | *Reference* |  |  | *Reference* |  |  |
| ICI-pneumonitis (all grade) |  |  | 0.010 |  |  | 0.003 |  |
| No | 145 | *Reference* |  |  | *Reference* |  |  |
| Yes | 6 | 3.66 (1.58-8.45) |  |  | 3.78 (1.57-9.11) |  |  |
| FEV_1_/FVC ratio |  |  | 0.754 |  |  |  |  |
| ≥0.7 | 118 | *Reference* |  |  |  |  |  |
| <0.7 | 33 | 0.93 (0.58-1.48) |  |  |  |  |  |
| FEV_1_ pred(%) |  |  | 0.003 |  |  | 0.006 |  |
| Preserved FEV_1_ (≥ 80%) | 86 | *Reference* |  |  | *Reference* |  |  |
| Reduced FEV_1_ (< 80%) | 65 | 1.76 (1.21-2.55) |  |  | 1.80 (1.18-2.74) |  |  |

Abbreviation: *ALK*: *anaplastic lymphoma kinase* gene rearrangement; Combination therapy: immune checkpoint inhibitors combined with other anticancer therapy, included chemotherapy, anti-angiogenesis, or tyrosine kinase inhibitor; ECOG PS: Eastern Cooperative Oncology Group performance status; *EGFR*: *epidermal growth factor receptor* mutation; FEV_1_: forced expiratory volume in 1 second; FEV_1_ pred(%): percentage of predicted FEV_1_; FVC: forced vital capacity; HR: hazard ratio; ICI: immune checkpoint inhibitors; ICI-pneumonitis: immune checkpoint inhibitor related pneumonitis; PD-L1: programmed cell death ligand-1; *ROS-1*: *cROS oncogene-1* rearrangement; WT: wild type of epidermal growth factor receptor.

**Supplementary Table 4: Cox regression model of OS in all patients received ICI (80% of predicted FEV_1_ as a cut-off value of FEV_1_)**

|  |  | **Univariate analysis** | |  | **Multivariable analysis(*P* <0.1)** | |  |
| --- | --- | --- | --- | --- | --- | --- | --- |
| **Variable** | **N** | **HR (95% CI)** | ***P* value** |  | **HR (95% CI)** | ***P* value** |  |
| Age at ICIs treatment |  |  | 0.860 |  |  |  |  |
| <70 yrs | 108 | *Reference* |  |  |  |  |  |
| ≥70 yrs | 43 | 0.96 (0.58-1.58) |  |  |  |  |  |
| Gender |  |  | 0.940 |  |  |  |  |
| Female | 49 | *Reference* |  |  |  |  |  |
| Male | 102 | 0.98 (0.61-1.57) |  |  |  |  |  |
| Smoking history |  |  | 0.730 |  |  |  |  |
| No | 68 | *Reference* |  |  |  |  |  |
| Yes | 83 | 1.08 (0.39-1.70) |  |  |  |  |  |
| ECOG PS |  |  | 0.004 |  |  | 0.013 |  |
| 0 | 61 | *Reference* |  |  | *Reference* |  |  |
| 1-2 | 90 | 2.05 (1.26-3.35) |  |  | 1.90 (1.14-3.16) |  |  |
| Stage |  |  | 0.063 |  |  | 0.037 |  |
| IVA | 58 | 0.64 (0.40-1.03) |  |  | 0.57 (0.34-0.97) |  |  |
| IVB & IVC | 93 | *Reference* |  |  | *Reference* |  |  |
| Initial Brain metastasis |  |  | 0.071 |  |  | 0.701 |  |
| No | 117 | *Reference* |  |  | *Reference* |  |  |
| Yes | 34 | 1.59 (0.96-2.64) |  |  | 1.11 (0.65-1.91) |  |  |
| Initial Lung metastasis |  |  | 0.964 |  |  |  |  |
| No | 101 | *Reference* |  |  |  |  |  |
| Yes | 50 | 1.01 (0.64-1.61) |  |  |  |  |  |
| Initial Liver metastasis |  |  | 0.026 |  |  | 0.049 |  |
| No | 134 | *Reference* |  |  | *Reference* |  |  |
| Yes | 17 | 2.08 (1.09-3.97) |  |  | 2.00 (1.00-4.00) |  |  |
| Pathology |  |  | 0.141 |  |  |  |  |
| Squamous cell carcinoma | 25 | *Reference* |  |  |  |  |  |
| Adenocarcinoma | 114 | 0.66 (0.38-1.15) |  |  |  |  |  |
| Known driver mutation |  |  | 0.253 |  |  |  |  |
| WT | 99 | *Reference* |  |  |  |  |  |
| *EGFR/ALK/ROS-1* (+) | 37 | 0.72 (0.41-1.26) |  |  |  |  |  |
| PD-L1 expression |  |  | 0.642 |  |  |  |  |
| <50% | 57 | *Reference* |  |  |  |  |  |
| ≥50% | 35 | 1.15 (0.63-2.11) |  |  |  |  |  |
| First-line therapy |  |  | 0.399 |  |  |  |  |
| No | 100 | *Reference* |  |  |  |  |  |
| Yes | 51 | 0.81 (0.49-1.33) |  |  |  |  |  |
| Prior radiotherapy |  |  | 0.074 |  |  | 0.112 |  |
| No | 62 | *Reference* |  |  | *Reference* |  |  |
| Yes | 89 | 1.53 (0.96-2.44) |  |  | 1.50 (0.91-2.47) |  |  |
| Prior lung surgery |  |  | 0.255 |  |  |  |  |
| No | 109 | *Reference* |  |  |  |  |  |
| Yes | 42 | 0.74 (0.44-1.24) |  |  |  |  |  |
| ICIs regimen |  |  | 0.035 |  |  | 0.060 |  |
| Monotherapy | 96 | 1.72 (1.04-2.83) |  |  | 1.65 (0.98-2.77) |  |  |
| Combination therapy | 55 | *Reference* |  |  | *Reference* |  |  |
| ICI-pneumonitis (all grade) |  |  | 0.087 |  |  | 0.025 |  |
| No | 145 | *Reference* |  |  | *Reference* |  |  |
| Yes | 6 | 2.42 (0.88-6.65) |  |  | 3.44 (1.17-10.09) |  |  |
| FEV_1_/FVC ratio |  |  | 0.478 |  |  |  |  |
| ≥0.7 | 118 | *Reference* |  |  |  |  |  |
| <0.7 | 33 | 1.22 (0.70-2.13) |  |  |  |  |  |
| FEV_1_ pred(%) |  |  | <0.001 |  |  | <0.001 |  |
| Preserved FEV_1_ (≥ 80%) | 86 | *Reference* |  |  | *Reference* |  |  |
| Reduced FEV_1_ (< 80%) | 65 | 2.44 (1.55-3.84) |  |  | 2.50 (1.56-3.99) |  |  |

Abbreviation: *ALK*: *anaplastic lymphoma kinase* gene rearrangement; Combination therapy: immune checkpoint inhibitors combined with other anticancer therapy, included chemotherapy, anti-angiogenesis, or tyrosine kinase inhibitor; ECOG PS: Eastern Cooperative Oncology Group performance status; *EGFR*: *epidermal growth factor receptor* mutation; FEV_1_: forced expiratory volume in 1 second; FEV_1_ pred(%): percentage of predicted FEV_1_; FVC: forced vital capacity; HR: hazard ratio; ICI: immune checkpoint inhibitors; ICI-pneumonitis: immune checkpoint inhibitor related pneumonitis; PD-L1: programmed cell death ligand-1; *ROS-1*: *cROS oncogene-1* rearrangement; WT: wild type of epidermal growth factor receptor.

**Supplementary Figure 1. Kaplan–Meier curve of PFS in all patients received ICI (4 groups of FEV_1_ as a cut-off value of FEV_1_)**

**
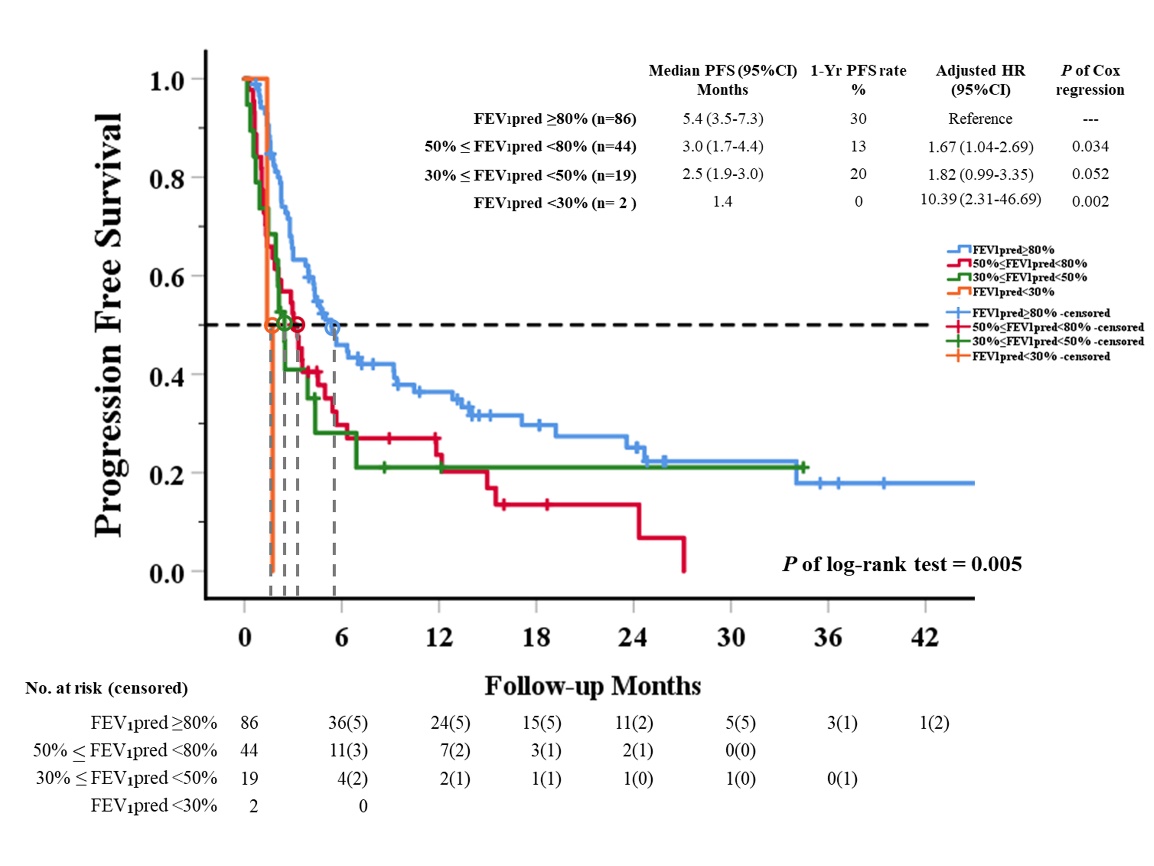
**

**Supplementary Table 5. Cox regression model of PFS in all patients received ICI (4 groups of FEV_1_ as a cut-off value of FEV_1_)**

|  |  | **Univariate analysis** | |  | **Multivariable analysis (*P* <0.1)** | |  |
| --- | --- | --- | --- | --- | --- | --- | --- |
| **Variable** | **N** | **HR (95% CI)** | ***P* value** |  | **HR (95% CI)** | ***P* value** |  |
| Age at ICIs treatment |  |  | 0.276 |  |  |  |  |
| <70 yrs | 108 | *Reference* |  |  |  |  |  |
| ≥70 yrs | 43 | 1.25 (0.84-1.87) |  |  |  |  |  |
| Gender |  |  | 0.046 |  |  | 0.091 |  |
| Female | 49 | *Reference* |  |  | *Reference* |  |  |
| Male | 102 | 0.68 (0.46-0.99) |  |  | 0.65 (0.40-1.07) |  |  |
| Smoking history |  |  | 0.011 |  |  | 0.760 |  |
| No | 68 | *Reference* |  |  | *Reference* |  |  |
| Yes | 83 | 0.62 (0.43-0.90) |  |  | 0.93 (0.58-1.50) |  |  |
| ECOG PS |  |  | 0.003 |  |  | <0.001 |  |
| 0 | 61 | *Reference* |  |  | *Reference* |  |  |
| 1-2 | 90 | 1.83 (1.24-2.71) |  |  | 2.21 (1.42-3.44) |  |  |
| Stage |  |  | 0.450 |  |  |  |  |
| IVA | 58 | 0.86 (0.59-1.26) |  |  |  |  |  |
| IVB & IVC | 93 | *Reference* |  |  |  |  |  |
| Initial Brain metastasis |  |  | 0.164 |  |  |  |  |
| No | 117 | *Reference* |  |  |  |  |  |
| Yes | 34 | 1.38 (0.88-2.12) |  |  |  |  |  |
| Initial Lung metastasis |  |  | 0.048 |  |  | 0.015 |  |
| No | 101 | *Reference* |  |  | *Reference* |  |  |
| Yes | 50 | 1.47 (1.00-2.14) |  |  | 1.69 (1.11-2.58) |  |  |
| Initial Liver metastasis |  |  | 0.020 |  |  | 0.003 |  |
| No | 134 | *Reference* |  |  | *Reference* |  |  |
| Yes | 17 | 1.91 (1.10-3.29) |  |  | 2.50 (1.37-4.58) |  |  |
| Pathology |  |  | 0.547 |  |  |  |  |
| Squamous cell carcinoma | 25 | *Reference* |  |  |  |  |  |
| Adenocarcinoma | 114 | 0.86 (0.53-1.39) |  |  |  |  |  |
| Known driver mutation |  |  | 0.125 |  |  |  |  |
| WT | 99 | *Reference* |  |  |  |  |  |
| *EGFR/ALK/ROS-1* (+) | 37 | 1.39 (0.91-2.11) |  |  |  |  |  |
| PD-L1 expression |  |  | 0.826 |  |  |  |  |
| <50% | 57 | *Reference* |  |  |  |  |  |
| ≥50% | 35 | 1.06 (0.63-1.78) |  |  |  |  |  |
| First-line therapy |  |  | 0.011 |  |  | 0.252 |  |
| No | 100 | *Reference* |  |  | *Reference* |  |  |
| Yes | 51 | 0.58 (0.38-0.89) |  |  | 0.73 (0.43-1.25) |  |  |
| Prior radiotherapy |  |  | 0.068 |  |  | 0.012 |  |
| No | 62 | *Reference* |  |  | *Reference* |  |  |
| Yes | 89 | 1.43 (0.98-2.09) |  |  | 1.70 (1.13-2.57) |  |  |
| Prior lung surgery |  |  | 0.199 |  |  |  |  |
| No | 109 | *Reference* |  |  |  |  |  |
| Yes | 42 | 0.75 (0.49-1.16) |  |  |  |  |  |
| ICIs regimen |  |  | 0.007 |  |  | 0.261 |  |
| Monotherapy | 96 | 1.73 (1.16-2.58) |  |  | 1.30 (0.82-2.06) |  |  |
| Combination therapy | 55 | *Reference* |  |  | *Reference* |  |  |
| ICI-pneumonitis (all grade) |  |  | 0.010 |  |  | 0.002 |  |
| No | 145 | *Reference* |  |  | *Reference* |  |  |
| Yes | 6 | 3.66 (1.58-8.45) |  |  | 3.94 (1.63-9.52) |  |  |
| FEV_1_/FVC ratio |  |  | 0.754 |  |  |  |  |
| ≥0.7 | 118 | *Reference* |  |  |  |  |  |
| <0.7 | 33 | 0.93 (0.58-1.48) |  |  |  |  |  |
| FEV_1_ pred(%) |  |  | 0.009 |  |  | 0.003 |  |
| FEV_1_ ≥ 80% | 86 | *Reference* |  |  | *Reference* |  |  |
| 50% ≤ FEV_1_ < 80% | 44 | 1.71 (1.13-2.58) |  |  | 1.67 (1.04-2.69) |  |  |
| 30% ≤ FEV_1_ < 50% | 19 | 1.73 (0.96-3.11) |  |  | 1.82 (0.99-3.35) |  |  |
| FEV_1_ < 30% | 2 | 5.39 (1.28-22.73) |  |  | 10.39 (2.31-46.69) |  |  |

Abbreviation: *ALK*: *anaplastic lymphoma kinase* gene rearrangement; Combination therapy: immune checkpoint inhibitors combined with other anticancer therapy, included chemotherapy, anti-angiogenesis, or tyrosine kinase inhibitor; ECOG PS: Eastern Cooperative Oncology Group performance status; *EGFR*: *epidermal growth factor receptor* mutation; FEV_1_: forced expiratory volume in 1 second; FEV_1_ pred(%): percentage of predicted FEV_1_; FVC: forced vital capacity; HR: hazard ratio; ICI: immune checkpoint inhibitors; ICI-pneumonitis: immune checkpoint inhibitor related pneumonitis; PD-L1: programmed cell death ligand-1; *ROS-1*: *cROS oncogene-1* rearrangement; WT: wild type of epidermal growth factor receptor.

**Supplementary Figure 2. Kaplan–Meier curve of OS in all patients received ICI (4 groups of FEV_1_ as a cut-off value of FEV_1_)**

**
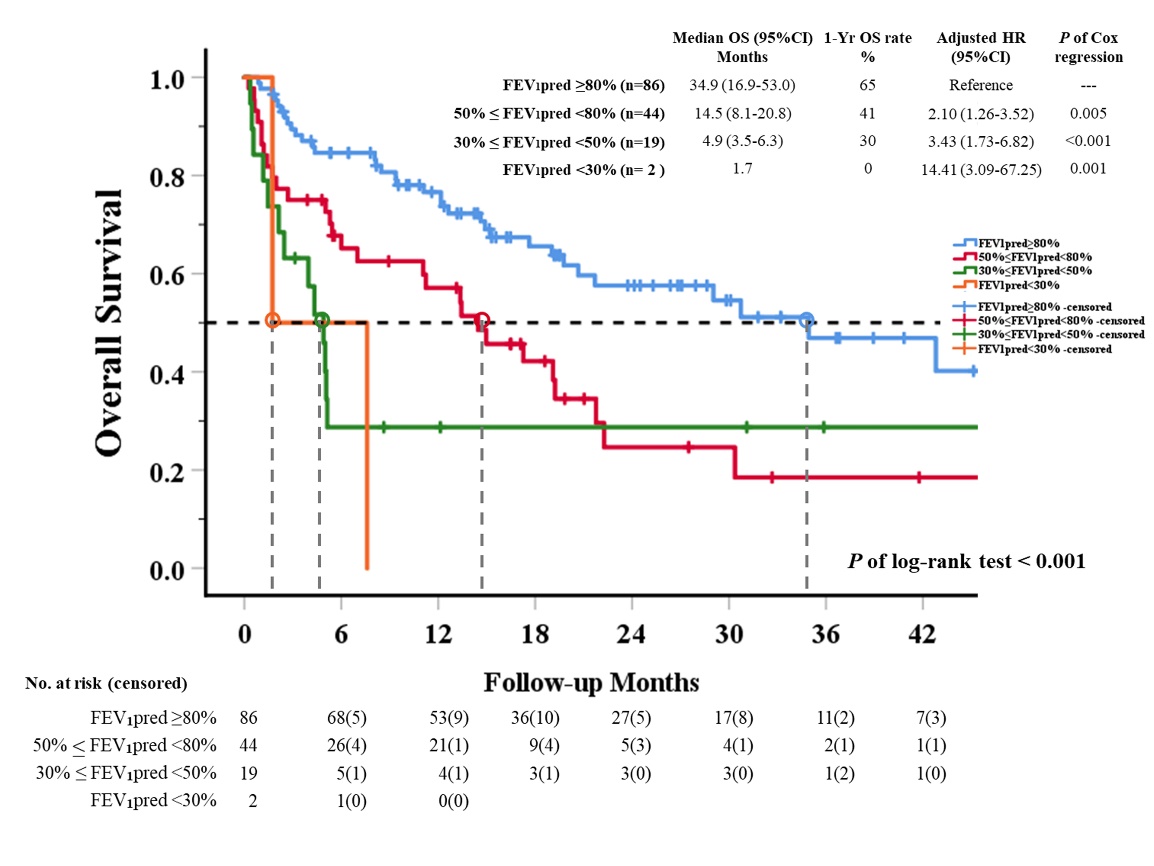
**

**Supplementary Table 6. Cox regression model of OS in all patients received ICI (4 groups of FEV_1_ as a cut-off value of FEV_1_)**

|  |  | **Univariate analysis** | |  | **Multivariable analysis (*P* <0.1)** | |  |
| --- | --- | --- | --- | --- | --- | --- | --- |
| **Variable** | **N** | **HR (95% CI)** | ***P* value** |  | **HR (95% CI)** | ***P* value** |  |
| Age at ICIs treatment |  |  | 0.860 |  |  |  |  |
| <70 yrs | 108 | *Reference* |  |  |  |  |  |
| ≥70 yrs | 43 | 0.96 (0.58-1.58) |  |  |  |  |  |
| Gender |  |  | 0.940 |  |  |  |  |
| Female | 49 | *Reference* |  |  |  |  |  |
| Male | 102 | 0.98 (0.61-1.57) |  |  |  |  |  |
| Smoking history |  |  | 0.730 |  |  |  |  |
| No | 68 | *Reference* |  |  |  |  |  |
| Yes | 83 | 1.08 (0.39-1.70) |  |  |  |  |  |
| ECOG PS |  |  | 0.004 |  |  | 0.009 |  |
| 0 | 61 | *Reference* |  |  | *Reference* |  |  |
| 1-2 | 90 | 2.05 (1.26-3.35) |  |  | 2.02 (1.20-3.41) |  |  |
| Stage |  |  | 0.063 |  |  | 0.032 |  |
| IVA | 58 | 0.64 (0.40-1.03) |  |  | 0.56 (0.33-0.95) |  |  |
| IVB & IVC | 93 | *Reference* |  |  | *Reference* |  |  |
| Initial Brain metastasis |  |  | 0.071 |  |  | 0.570 |  |
| No | 117 | *Reference* |  |  | *Reference* |  |  |
| Yes | 34 | 1.59 (0.96-2.64) |  |  | 1.17 (0.68-2.04) |  |  |
| Initial Lung metastasis |  |  | 0.964 |  |  |  |  |
| No | 101 | *Reference* |  |  |  |  |  |
| Yes | 50 | 1.01 (0.64-1.61) |  |  |  |  |  |
| Initial Liver metastasis |  |  | 0.026 |  |  | 0.030 |  |
| No | 134 | *Reference* |  |  | *Reference* |  |  |
| Yes | 17 | 2.08 (1.09-3.97) |  |  | 2.15 (1.08-4.30) |  |  |
| Pathology |  |  | 0.141 |  |  |  |  |
| Squamous cell carcinoma | 25 | *Reference* |  |  |  |  |  |
| Adenocarcinoma | 114 | 0.66 (0.38-1.15) |  |  |  |  |  |
| Known driver mutation |  |  | 0.253 |  |  |  |  |
| WT | 99 | *Reference* |  |  |  |  |  |
| *EGFR/ALK/ROS-1* (+) | 37 | 0.72 (0.41-1.26) |  |  |  |  |  |
| PD-L1 expression |  |  | 0.642 |  |  |  |  |
| <50% | 57 | *Reference* |  |  |  |  |  |
| ≥50% | 35 | 1.15 (0.63-2.11) |  |  |  |  |  |
| First-line therapy |  |  | 0.399 |  |  |  |  |
| No | 100 | *Reference* |  |  |  |  |  |
| Yes | 51 | 0.81 (0.49-1.33) |  |  |  |  |  |
| Prior radiotherapy |  |  | 0.074 |  |  | 0.268 |  |
| No | 62 | *Reference* |  |  | *Reference* |  |  |
| Yes | 89 | 1.53 (0.96-2.44) |  |  | 1.34 (0.80-2.25) |  |  |
| Prior lung surgery |  |  | 0.255 |  |  |  |  |
| No | 109 | *Reference* |  |  |  |  |  |
| Yes | 42 | 0.74 (0.44-1.24) |  |  |  |  |  |
| ICIs regimen |  |  | 0.035 |  |  | 0.042 |  |
| Monotherapy | 96 | 1.72 (1.04-2.83) |  |  | 1.73 (1.02-2.93) |  |  |
| Combination therapy | 55 | *Reference* |  |  | *Reference* |  |  |
| ICI-pneumonitis (all grade) |  |  | 0.087 |  |  | 0.018 |  |
| No | 145 | *Reference* |  |  | *Reference* |  |  |
| Yes | 6 | 2.42 (0.88-6.65) |  |  | 3.72 (1.26-11.02) |  |  |
| FEV_1_/FVC ratio |  |  | 0.478 |  |  |  |  |
| ≥0.7 | 118 | *Reference* |  |  |  |  |  |
| <0.7 | 33 | 1.22 (0.70-2.13) |  |  |  |  |  |
| FEV_1_ pred(%) |  |  | <0.001 |  |  | <0.001 |  |
| FEV_1_ ≥ 80% | 86 | *Reference* |  |  | *Reference* |  |  |
| 50% ≤ FEV_1_ < 80% | 44 | 2.13 (1.29-3.53) |  |  | 2.10 (1.26-3.52) |  |  |
| 30% ≤ FEV_1_ < 50% | 19 | 3.13 (1.64-5.97) |  |  | 3.43 (1.73-6.82) |  |  |
| FEV_1_ < 30% | 2 | 6.71 (1.58-28.48) |  |  | 14.41 (3.09-67.25) |  |  |

Abbreviation: *ALK*: *anaplastic lymphoma kinase* gene rearrangement; Combination therapy: immune checkpoint inhibitors combined with other anticancer therapy, included chemotherapy, anti-angiogenesis, or tyrosine kinase inhibitor; ECOG PS: Eastern Cooperative Oncology Group performance status; *EGFR*: *epidermal growth factor receptor* mutation; FEV_1_: forced expiratory volume in 1 second; FEV_1_ pred(%): percentage of predicted FEV_1_; FVC: forced vital capacity; HR: hazard ratio; ICI: immune checkpoint inhibitors; ICI-pneumonitis: immune checkpoint inhibitor related pneumonitis; PD-L1: programmed cell death ligand-1; *ROS-1*: *cROS oncogene-1* rearrangement; WT: wild type of epidermal growth factor receptor.

**Supplementary Figure 3: Kaplan–Meier curve of PFS in patients received first-line ICI**

**
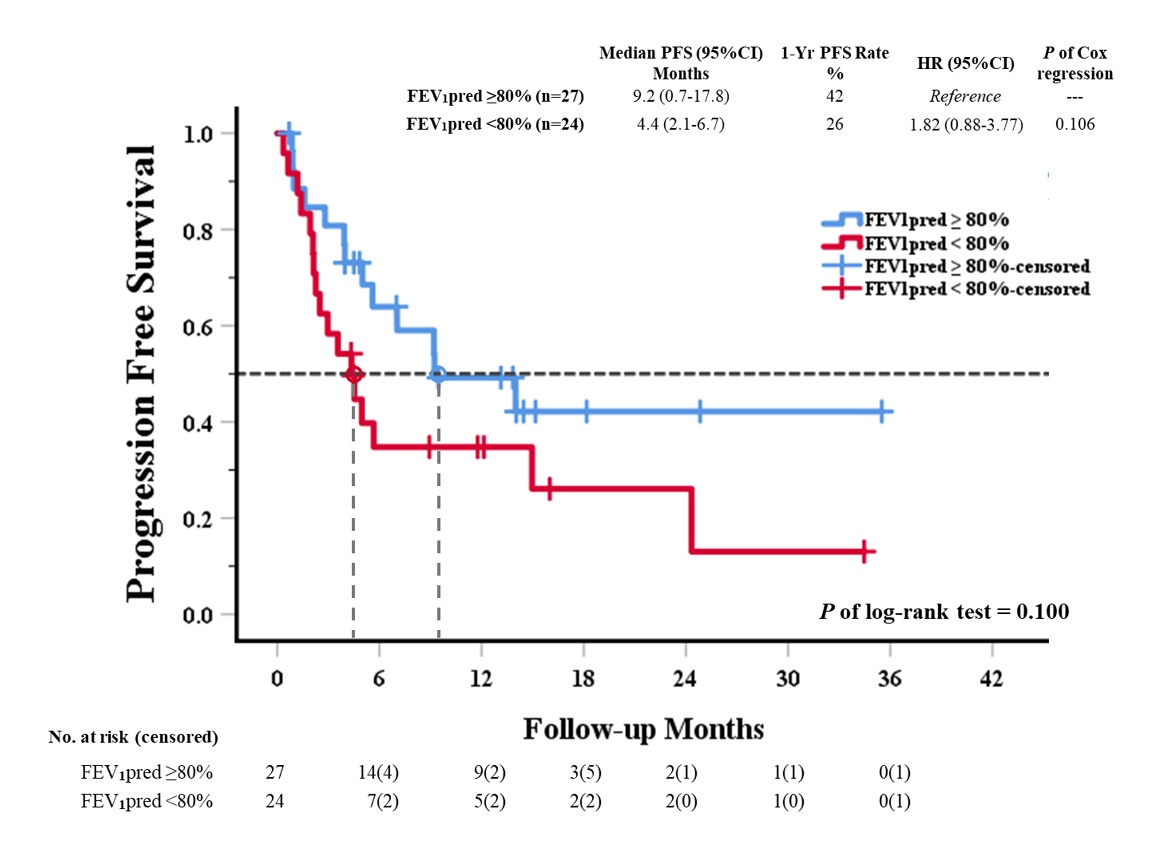
**

**Supplementary Table 7: Cox regression model of PFS in patients received first-line ICI**

|  |  | **Univariate analysis** | |  | **Multivariable analysis(*P*<0.1)** | |  |
| --- | --- | --- | --- | --- | --- | --- | --- |
| **Variable** | **N** | **HR (95% CI)** | ***P* value** |  | **HR (95% CI)** | ***P* value** |  |
| Age at ICIs treatment |  |  | 0.086 |  |  | 0.040 |  |
| <70 yrs | 33 | *Reference* |  |  | *Reference* |  |  |
| ≥70 yrs | 18 | 1.90 (0.91-3.93) |  |  | 2.52 (1.05-6.07) |  |  |
| Gender |  |  | 0.022 |  |  | 0.233 |  |
| Female | 13 | *Reference* |  |  | *Reference* |  |  |
| Male | 38 | 0.41 (0.19-0.88) |  |  | 0.27 (0.03-2.32) |  |  |
| Smoking history |  |  | 0.038 |  |  | 0.658 |  |
| No | 14 | *Reference* |  |  | *Reference* |  |  |
| Yes | 37 | 0.44 (0.20-0.96) |  |  | 0.61 (0.07-5.47) |  |  |
| ECOG PS |  |  | 0.069 |  |  | 0.138 |  |
| 0 | 21 | *Reference* |  |  | *Reference* |  |  |
| 1-2 | 30 | 2.05 (0.95-4.45) |  |  | 2.10 (0.79-5.61) |  |  |
| Stage |  |  | 0.529 |  |  |  |  |
| IVA | 20 | 0.78 (0.37-1.67) |  |  |  |  |  |
| IVB & IVC | 31 | *Reference* |  |  |  |  |  |
| Initial Brain metastasis |  |  | 0.016 |  |  | 0.041 |  |
| No | 36 | *Reference* |  |  | *Reference* |  |  |
| Yes | 15 | 2.63 (1.20-5.75) |  |  | 2.55 (1.04-6.26) |  |  |
| Initial Lung metastasis |  |  | 0.251 |  |  |  |  |
| No | 35 | *Reference* |  |  |  |  |  |
| Yes | 16 | 1.53 (0.74-3.17) |  |  |  |  |  |
| Initial Liver metastasis |  |  | 0.129 |  |  | 0.167 |  |
| No | 44 | *Reference* |  |  | *Reference* |  |  |
| Yes | 7 | 2.12 (0.80-5.57) |  |  | 2.30 (0.71-7.48) |  |  |
| Pathology |  |  | 0.185 |  |  |  |  |
| Squamous cell carcinoma | 7 | *Reference* |  |  |  |  |  |
| Adenocarcinoma | 39 | 0.53 (0.21-1.35) |  |  |  |  |  |
| PD-L1 expression |  |  | 0.085 |  |  |  |  |
| <50% | 20 | *Reference* |  |  |  |  |  |
| ≥50% | 19 | 2.24 (0.89-5.59) |  |  |  |  |  |
| Prior radiotherapy |  |  | 0.177 |  |  |  |  |
| No | 24 | *Reference* |  |  |  |  |  |
| Yes | 27 | 1.66 (0.80-3.46) |  |  |  |  |  |
| Prior lung surgery |  |  | 0.227 |  |  |  |  |
| No | 41 | *Reference* |  |  |  |  |  |
| Yes | 10 | 0.52 (0.18-1.50) |  |  |  |  |  |
| ICIs regimen |  |  | 0.040 |  |  | 0.043 |  |
| Monotherapy | 20 | 2.13 (1.03-4.39) |  |  | 2.44 (1.03-5.80) |  |  |
| Combination therapy | 31 | *Reference* |  |  | *Reference* |  |  |
| ICI-pneumonitis (all grade) |  |  | 0.009 |  |  | 0.002 |  |
| No | 50 | *Reference* |  |  |  |  |  |
| Yes | 1 | 24.32 (2.21-268.28) |  |  | 114.97 (5.36-2467.26) |  |  |
| FEV_1_/FVC ratio |  |  | 0.963 |  | *Reference* |  |  |
| ≥0.7 | 35 | *Reference* |  |  |  |  |  |
| <0.7 | 16 | 1.02 (0.48-2.18) |  |  |  |  |  |
| FEV_1_ pred(%) |  |  | 0.105 |  |  | 0.166 |  |
| FEV_1_ ≥ 80% | 27 | *Reference* |  |  | *Reference* |  |  |
| FEV_1_ < 80% | 24 | 1.82 (0.88-3.77) |  |  | 1.81 (0.78-4.19) |  |  |

Abbreviation: Combination therapy: immune checkpoint inhibitors combined with other anticancer therapy, included chemotherapy, anti-angiogenesis, or tyrosine kinase inhibitor; ECOG PS: Eastern Cooperative Oncology Group performance status; FEV_1_: forced expiratory volume in 1 second; FEV_1_ pred(%): percentage of predicted FEV_1_;FVC: forced vital capacity; ICI: immune checkpoint inhibitors; OR: odds ratio; ICI-pneumonitis: immune checkpoint inhibitor related pneumonitis; PD-L1: programmed cell death ligand-1.

**Supplementary Figure 4: Kaplan–Meier curve of OS in patients received first-line ICI**

**
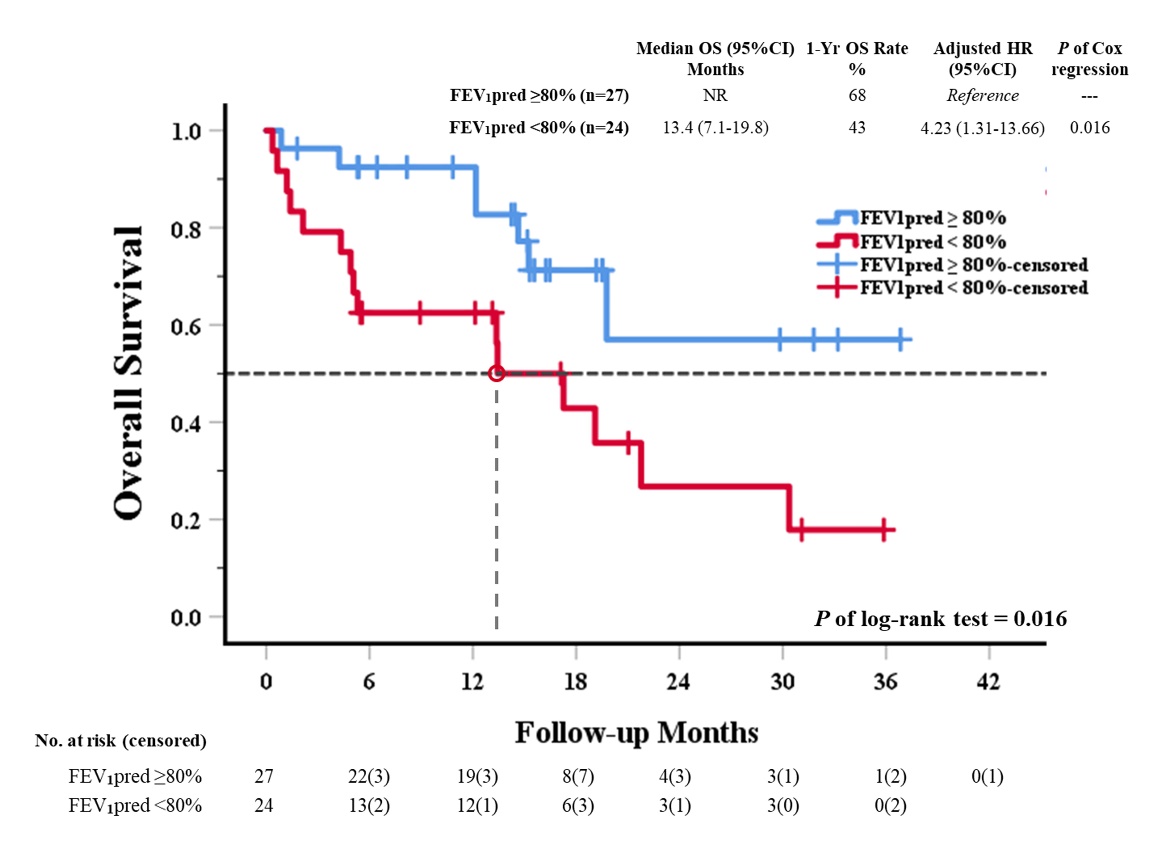
**

**Supplementary Table 8: Cox regression model of OS in patients received first-line ICI**

|  |  | **Univariate analysis** | |  | **Multivariable analysis(*P*<0.1)** | |  |
| --- | --- | --- | --- | --- | --- | --- | --- |
| **Variable** | **N** | **HR (95% CI)** | ***P* value** |  | **HR (95% CI)** | ***P* value** |  |
| Age at ICIs treatment |  |  | 0.291 |  |  |  |  |
| <70 yrs | 33 | *Reference* |  |  |  |  |  |
| ≥70 yrs | 18 | 1.59 (0.67-3.74) |  |  |  |  |  |
| Gender |  |  | 0.715 |  |  |  |  |
| Female | 13 | *Reference* |  |  |  |  |  |
| Male | 38 | 0.85 (0.34-2.09) |  |  |  |  |  |
| Smoking history |  |  | 0.788 |  |  |  |  |
| No | 14 | *Reference* |  |  |  |  |  |
| Yes | 37 | 0.88 (0.36-2.18) |  |  |  |  |  |
| ECOG PS |  |  | 0.005 |  |  | 0.082 |  |
| 0 | 21 | *Reference* |  |  | *Reference* |  |  |
| 1-2 | 30 | 4.37 (1.57-12.13) |  |  | 2.43 (0.89-6.59) |  |  |
| Stage |  |  | 0.034 |  |  | 0.016 |  |
| IVA | 20 | 0.36 (0.14-0.93) |  |  | 0.20 (0.05-0.74) |  |  |
| IVB & IVC | 31 | *Reference* |  |  | *Reference* |  |  |
| Initial Brain metastasis |  |  | 0.040 |  |  | 0.385 |  |
| No | 36 | *Reference* |  |  | *Reference* |  |  |
| Yes | 15 | 2.46 (1.04-5.82) |  |  | 1.56 (0.57-4.22) |  |  |
| Initial Lung metastasis |  |  | 0.832 |  |  |  |  |
| No | 35 | *Reference* |  |  |  |  |  |
| Yes | 16 | 0.91 (0.38-2.19) |  |  |  |  |  |
| Initial Liver metastasis |  |  | 0.024 |  |  | 0.231 |  |
| No | 44 | *Reference* |  |  | *Reference* |  |  |
| Yes | 7 | 3.24(1.17-8.95) |  |  | 2.44 (0.57-10.51) |  |  |
| Pathology |  |  | 0.287 |  |  |  |  |
| Squamous cell carcinoma | 7 | *Reference* |  |  |  |  |  |
| Adenocarcinoma | 39 | 0.55 (0.18-1.66) |  |  |  |  |  |
| PD-L1 expression |  |  | 0.082 |  |  |  |  |
| <50% | 20 | *Reference* |  |  |  |  |  |
| ≥50% | 19 | 2.69 (0.88-8.17) |  |  |  |  |  |
| Prior radiotherapy |  |  | 0.656 |  |  |  |  |
| No | 24 | *Reference* |  |  |  |  |  |
| Yes | 27 | 1.21 (0.52-2.82) |  |  |  |  |  |
| Prior lung surgery |  |  | 0.454 |  |  |  |  |
| No | 41 | *Reference* |  |  |  |  |  |
| Yes | 10 | 0.63 (0.19-2.13) |  |  |  |  |  |
| ICIs regimen |  |  | 0.089 |  |  | 0.118 |  |
| Monotherapy | 20 | 2.08 (0.90-4.81) |  |  | 2.54 (0.79-13.66) |  |  |
| Combination therapy | 31 | *Reference* |  |  | *Reference* |  |  |
| ICI-pneumonitis (all grade) |  |  | 0.009 |  |  | 0.035 |  |
| No | 50 | *Reference* |  |  | *Reference* |  |  |
| Yes | 1 | 24.50 (2.22-270.16) |  |  | 31.43 (1.28-771.07) |  |  |
| FEV_1_/FVC ratio |  |  | 0.627 |  |  |  |  |
| ≥0.7 | 35 | *Reference* |  |  |  |  |  |
| <0.7 | 16 | 1.25 (0.51-3.08) |  |  |  |  |  |
| FEV_1_ pred(%) |  |  | 0.021 |  |  | 0.016 |  |
| FEV_1_ ≥ 80% | 27 | *Reference* |  |  | *Reference* |  |  |
| FEV_1_ < 80% | 24 | 2.89 (1.17-7.11) |  |  | 4.23 (1.31-13.66) |  |  |

Abbreviation: Combination therapy: immune checkpoint inhibitors combined with other anticancer therapy, included chemotherapy, anti-angiogenesis, or tyrosine kinase inhibitor; ECOG PS: Eastern Cooperative Oncology Group performance status; FEV_1_: forced expiratory volume in 1 second; FEV_1_ pred(%): percentage of predicted FEV_1_;FVC: forced vital capacity; ICI: immune checkpoint inhibitors; OR: odds ratio; ICI-pneumonitis: immune checkpoint inhibitor related pneumonitis; PD-L1: programmed cell death ligand-1.

**Supplementary Figure 5: Kaplan–Meier curve of PFS in patients received ≥second-line ICI**

**
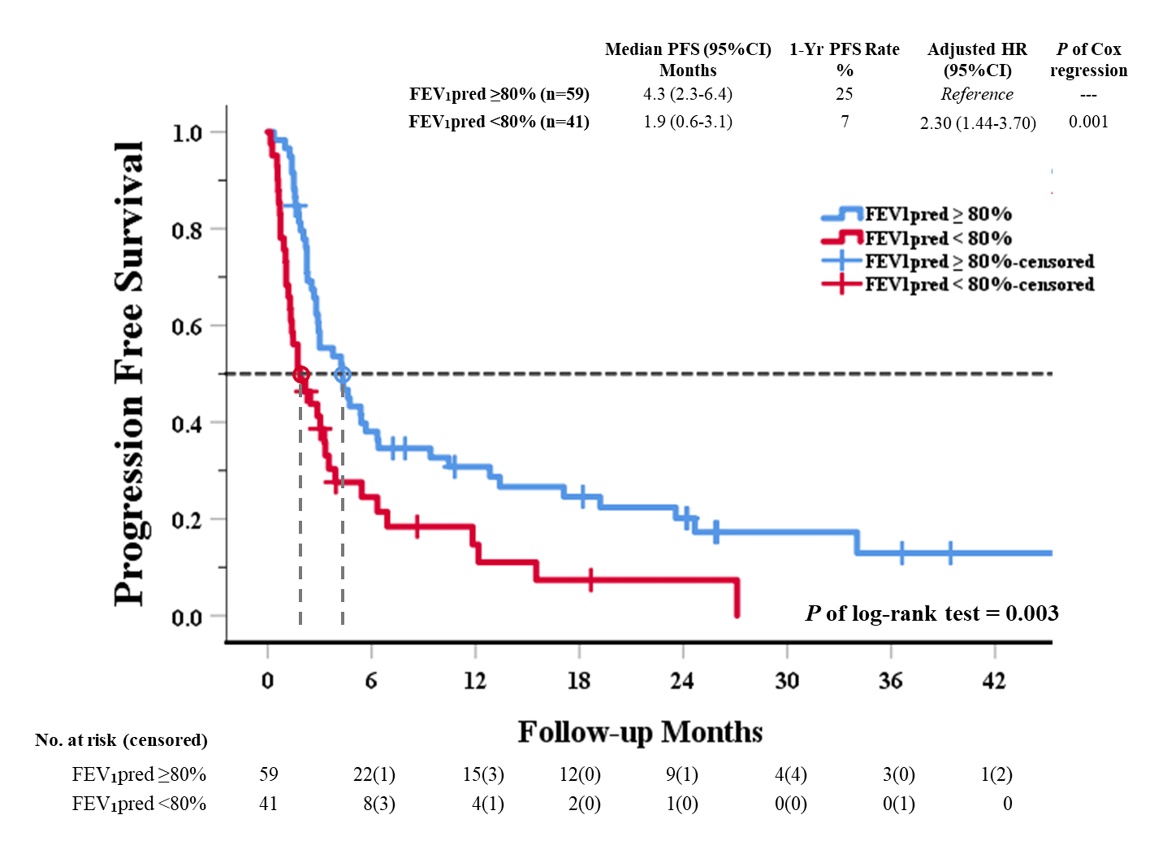
**

**Supplementary Table 9: Cox regression model of PFS in patients received ≥second-line ICI**

|  |  | **Univariate analysis** | |  | **Multivariable analysis(*P*<0.1)** | |  |
| --- | --- | --- | --- | --- | --- | --- | --- |
| **Variable** | **N** | **HR (95% CI)** | ***P* value** |  | **HR (95% CI)** | ***P* value** |  |
| Age at ICIs treatment |  |  | 0.631 |  |  |  |  |
| <70 yrs | 75 | *Reference* |  |  |  |  |  |
| ≥70 yrs | 25 | 1.13 (0.69-1.86) |  |  |  |  |  |
| Gender |  |  | 0.590 |  |  |  |  |
| Female | 36 | *Reference* |  |  |  |  |  |
| Male | 64 | 0.88 (0.57-1.38) |  |  |  |  |  |
| Smoking history |  |  | 0.386 |  |  |  |  |
| No | 54 | *Reference* |  |  |  |  |  |
| Yes | 46 | 0.82 (0.53-1.28) |  |  |  |  |  |
| ECOG PS |  |  | 0.019 |  |  | 0.041 |  |
| 0 | 40 | *Reference* |  |  | *Reference* |  |  |
| 1-2 | 60 | 1.72 (1.09-2.71) |  |  | 1.63 (1.02-2.62) |  |  |
| Stage |  |  | 0.758 |  |  |  |  |
| IVA | 38 | 0.93 (0.60-1.45) |  |  |  |  |  |
| IVB & IVC | 62 | *Reference* |  |  |  |  |  |
| Initial Brain metastasis |  |  | 0.758 |  |  |  |  |
| No | 81 | *Reference* |  |  |  |  |  |
| Yes | 19 | 1.09 (0.62-1.92) |  |  |  |  |  |
| Initial Lung metastasis |  |  | 0.095 |  |  | 0.027 |  |
| No | 66 | *Reference* |  |  | *Reference* |  |  |
| Yes | 34 | 1.46 (0.17-1.23) |  |  | 1.73 (1.07-2.81) |  |  |
| Initial Liver metastasis |  |  | 0.059 |  |  | 0.055 |  |
| No | 90 | *Reference* |  |  | *Reference* |  |  |
| Yes | 10 | 1.91 (0.98-3.71) |  |  | 1.95 (0.99-3.85) |  |  |
| Pathology |  |  | 0.802 |  |  |  |  |
| Squamous cell carcinoma | 18 | *Reference* |  |  |  |  |  |
| Adenocarcinoma | 75 | 1.08 (0.61-1.90) |  |  |  |  |  |
| PD-L1 expression |  |  | 0.472 |  |  |  |  |
| <50% | 37 | *Reference* |  |  |  |  |  |
| ≥50% | 16 | 0.78 (0.40-1.53) |  |  |  |  |  |
| Prior radiotherapy |  |  | 0.257 |  |  |  |  |
| No | 38 | *Reference* |  |  |  |  |  |
| Yes | 62 | 1.29 (0.83-2.02) |  |  |  |  |  |
| Prior lung surgery |  |  | 0.242 |  |  |  |  |
| No | 68 | *Reference* |  |  |  |  |  |
| Yes | 32 | 0.75 (0.46-1.22) |  |  |  |  |  |
| ICIs regimen |  |  | 0.476 |  |  |  |  |
| Monotherapy | 76 | 1.21 (0.72-2.02) |  |  |  |  |  |
| Combination therapy | 24 | *Reference* |  |  |  |  |  |
| ICI-pneumonitis (all grade) |  |  | 0.040 |  |  | 0.004 |  |
| No | 95 | *Reference* |  |  | *Reference* |  |  |
| Yes | 5 | 2.63 (1.05-6.58) |  |  | 3.99 (1.54-10.34) |  |  |
| FEV_1_/FVC ratio |  |  | 0.822 |  |  |  |  |
| ≥0.7 | 83 | *Reference* |  |  |  |  |  |
| <0.7 | 17 | 1.07 (0.58-1.99) |  |  |  |  |  |
| FEV_1_ pred(%) |  |  | 0.003 |  |  | 0.001 |  |
| FEV_1_ ≥ 80% | 59 | *Reference* |  |  | *Reference* |  |  |
| FEV_1_ < 80% | 41 | 1.95 (1.25-3.03) |  |  | 2.30 (1.44-3.70) |  |  |

Abbreviation: Combination therapy: immune checkpoint inhibitors combined with other anticancer therapy, included chemotherapy, anti-angiogenesis, or tyrosine kinase inhibitor; ECOG PS: Eastern Cooperative Oncology Group performance status; FEV_1_: forced expiratory volume in 1 second; FEV_1_ pred(%): percentage of predicted FEV_1_;FVC: forced vital capacity; ICI: immune checkpoint inhibitors; OR: odds ratio; ICI-pneumonitis: immune checkpoint inhibitor related pneumonitis; PD-L1: programmed cell death ligand-1.

**Supplementary Figure 9: Kaplan–Meier curve of OS in patients received ≥second-line ICI**

**
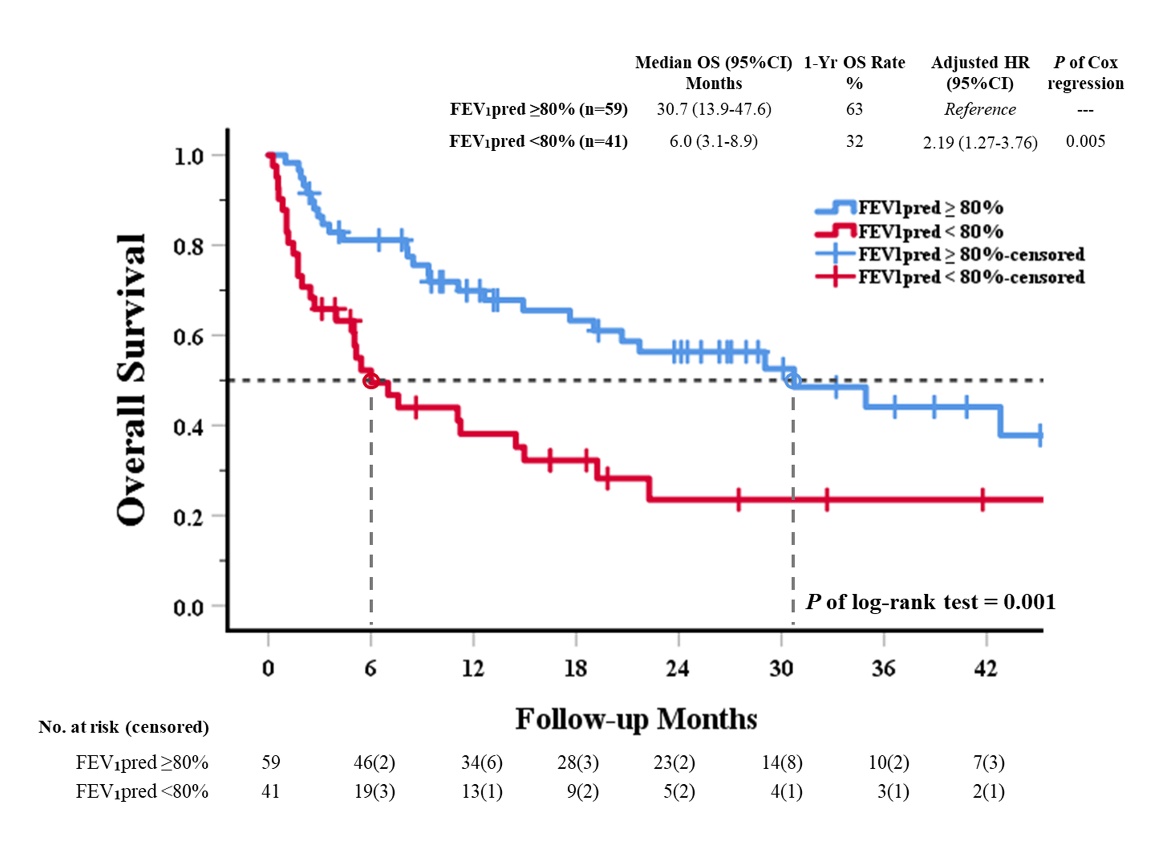
**

**Supplementary Table 10: Cox regression model of OS in patients received ≥second-line ICI**

|  |  | **Univariate analysis** | |  | **Multivariable analysis(*P*<0.1)** | |  |
| --- | --- | --- | --- | --- | --- | --- | --- |
| **Variable** | **N** | **HR (95% CI)** | ***P* value** |  | **HR (95% CI)** | ***P* value** |  |
| Age at ICIs treatment |  |  | 0.469 |  |  |  |  |
| <70 yrs | 75 | *Reference* |  |  |  |  |  |
| ≥70 yrs | 25 | 0.79 (0.41-1.50) |  |  |  |  |  |
| Gender |  |  | 0.771 |  |  |  |  |
| Female | 36 | *Reference* |  |  |  |  |  |
| Male | 64 | 1.09 (0.63-1.88) |  |  |  |  |  |
| Smoking history |  |  | 0.350 |  |  |  |  |
| No | 54 | *Reference* |  |  |  |  |  |
| Yes | 46 | 1.29 (0.76-2.20) |  |  |  |  |  |
| ECOG PS |  |  | 0.105 |  |  | 0.209 |  |
| 0 | 40 | *Reference* |  |  | *Reference* |  |  |
| 1-2 | 60 | 1.59 (0.91-2.80) |  |  | 1.45 (0.81-2.57) |  |  |
| Stage |  |  | 0.361 |  |  |  |  |
| IVa | 38 | 0.77 (0.44-1.35) |  |  |  |  |  |
| IVb & IVc | 62 | *Reference* |  |  |  |  |  |
| Initial Brain metastasis |  |  | 0.412 |  |  |  |  |
| No | 81 | *Reference* |  |  |  |  |  |
| Yes | 19 | 1.31 (0.69-2.49) |  |  |  |  |  |
| Initial Lung metastasis |  |  | 0.956 |  |  |  |  |
| No | 66 | *Reference* |  |  |  |  |  |
| Yes | 34 | 1.02 (0.59-1.76) |  |  |  |  |  |
| Initial Liver metastasis |  |  | 0.348 |  |  |  |  |
| No | 90 | *Reference* |  |  |  |  |  |
| Yes | 10 | 1.51 (0.64-3.55) |  |  |  |  |  |
| Pathology |  |  | 0.285 |  |  |  |  |
| Squamous cell carcinoma | 18 | *Reference* |  |  |  |  |  |
| Adenocarcinoma | 75 | 0.70 (0.37-1.34) |  |  |  |  |  |
| PD-L1 expression |  |  | 0.681 |  |  |  |  |
| <50% | 37 | *Reference* |  |  |  |  |  |
| ≥50% | 16 | 0.85 (0.38-1.89) |  |  |  |  |  |
| Prior radiotherapy |  |  | 0.082 |  |  | 0.077 |  |
| No | 38 | *Reference* |  |  | *Reference* |  |  |
| Yes | 62 | 1.66 (0.94-2.92) |  |  | 1.67 (0.95-2.96) |  |  |
| Prior lung surgery |  |  | 0.349 |  |  |  |  |
| No | 68 | *Reference* |  |  |  |  |  |
| Yes | 32 | 0.76 (0.42-1.36) |  |  |  |  |  |
| ICIs regimen |  |  | 0.280 |  |  |  |  |
| Monotherapy | 76 | 1.44 (0.74-2.79) |  |  |  |  |  |
| Combination therapy | 24 | *Reference* |  |  |  |  |  |
| ICI-pneumonitis (all grade) |  |  | 0.399 |  |  |  |  |
| No | 95 | *Reference* |  |  |  |  |  |
| Yes | 5 | 1.65 (0.51-5.32) |  |  |  |  |  |
| FEV_1_/FVC ratio |  |  | 0.394 |  |  |  |  |
| ≥0.7 | 83 | *Reference* |  |  |  |  |  |
| <0.7 | 17 | 1.37 (0.67-2.81) |  |  |  |  |  |
| FEV_1_ pred(%) |  |  | 0.002 |  |  | 0.005 |  |
| FEV_1_ ≥ 80% | 59 | *Reference* |  |  | *Reference* |  |  |
| FEV_1_ < 80% | 41 | 2.34 (1.37-3.99) |  |  | 2.19 (1.27-3.76) |  |  |

Abbreviation: Combination therapy: immune checkpoint inhibitors combined with other anticancer therapy, included chemotherapy, anti-angiogenesis, or tyrosine kinase inhibitor; ECOG PS: Eastern Cooperative Oncology Group performance status; FEV_1_: forced expiratory volume in 1 second; FEV_1_ pred(%): percentage of predicted FEV_1_;FVC: forced vital capacity; ICI: immune checkpoint inhibitors; OR: odds ratio; ICI-pneumonitis: immune checkpoint inhibitor related pneumonitis; PD-L1: programmed cell death ligand-1.

**Supplementary Figure 7: Kaplan–Meier curve of PFS in patients received ICI without driver mutation**

**
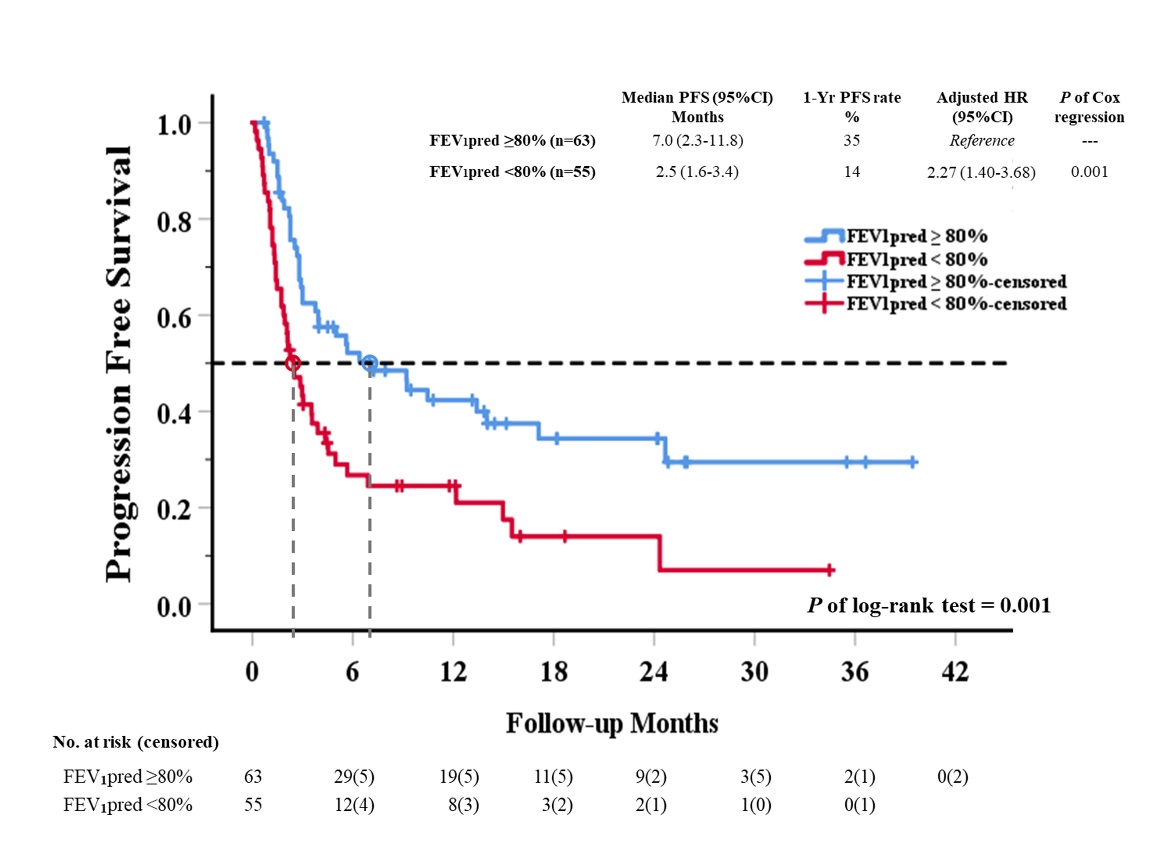
**

**Supplementary Table 11: Cox regression model of PFS in patients received ICI without driver mutation**

|  |  | **Univariate analysis** | |  | **Multivariable analysis(*P*<0.1)** | |  |
| --- | --- | --- | --- | --- | --- | --- | --- |
| **Variable** | **N** | **HR (95% CI)** | ***P* value** |  | **HR (95% CI)** | ***P* value** |  |
| Age at ICIs treatment |  |  | 0.405 |  |  |  |  |
| <70 yrs | 83 | *Reference* |  |  |  |  |  |
| ≥70 yrs | 35 | 1.22 (0.77-1.94) |  |  |  |  |  |
| Gender |  |  | 0.128 |  |  |  |  |
| Female | 34 | *Reference* |  |  |  |  |  |
| Male | 84 | 0.70 (0.44-1.11) |  |  |  |  |  |
| Smoking history |  |  | 0.010 |  |  | 0.061 |  |
| No | 43 | *Reference* |  |  | *Reference* |  |  |
| Yes | 75 | 0.56 (0.36-0.87) |  |  | 0.61 (0.37-1.02) |  |  |
| ECOG PS |  |  | <0.001 |  |  | 0.004 |  |
| 0 | 47 | *Reference* |  |  | *Reference* |  |  |
| 1-2 | 71 | 2.36 (1.46-3.81) |  |  | 2.12 (1.28-3.54) |  |  |
| Stage |  |  | 0.548 |  |  |  |  |
| IVA | 45 | 0.87 (0.56-1.36) |  |  |  |  |  |
| IVB & IVC | 73 | *Reference* |  |  |  |  |  |
| Initial Brain metastasis |  |  | 0.371 |  |  |  |  |
| No | 91 | *Reference* |  |  |  |  |  |
| Yes | 27 | 1.27 (0.76-2.12) |  |  |  |  |  |
| Initial Lung metastasis |  |  | 0.062 |  |  | 0.032 |  |
| No | 82 | *Reference* |  |  | *Reference* |  |  |
| Yes | 36 | 1.54 (0.98-2.41) |  |  | 1.72 (1.05-2.83) |  |  |
| Initial Liver metastasis |  |  | 0.017 |  |  | 0.001 |  |
| No | 105 | *Reference* |  |  | *Reference* |  |  |
| Yes | 13 | 2.19 (1.15-4.16) |  |  | 3.11 (1.57-6.15) |  |  |
| Pathology |  |  | 0.201 |  |  |  |  |
| Squamous cell carcinoma | 20 | *Reference* |  |  |  |  |  |
| Adenocarcinoma | 86 | 0.70 (0.41-1.21) |  |  |  |  |  |
| PD-L1 expression |  |  | 0.935 |  |  |  |  |
| <50% | 51 | *Reference* |  |  |  |  |  |
| ≥50% | 33 | 1.02 (0.59-1.77) |  |  |  |  |  |
| First-line therapy |  |  | 0.012 |  |  | 0.180 |  |
| No | 68 | *Reference* |  |  | *Reference* |  |  |
| Yes | 50 | 0.56 (0.36-0.88) |  |  | 0.66 (0.36-1.21) |  |  |
| Prior radiotherapy |  |  | 0.080 |  |  | 0.008 |  |
| No | 47 | *Reference* |  |  | *Reference* |  |  |
| Yes | 71 | 1.50 (0.95-2.36) |  |  | 1.98 (1.20-3.28) |  |  |
| Prior lung surgery |  |  | 0.232 |  |  |  |  |
| No | 82 | *Reference* |  |  |  |  |  |
| Yes | 36 | 0.74 (0.46-1.21) |  |  |  |  |  |
| ICIs regimen |  |  | 0.002 |  |  | 0.262 |  |
| Monotherapy | 74 | 2.15 (1.33-3.47) |  |  | 1.39 (0.78-2.48) |  |  |
| Combination therapy | 44 | *Reference* |  |  | *Reference* |  |  |
| ICI-pneumonitis (all grade) |  |  | 0.001 |  |  | 0.001 |  |
| No | 113 | *Reference* |  |  | *Reference* |  |  |
| Yes | 5 | 5.22 (2.05-13.30) |  |  | 5.02 (1.86-13.54) |  |  |
| FEV_1_/FVC ratio |  |  | 0.967 |  |  |  |  |
| ≥0.7 | 118 | *Reference* |  |  |  |  |  |
| <0.7 | 33 | 0.99 (0.61-1.62) |  |  |  |  |  |
| FEV_1_ pred(%) |  |  | 0.001 |  |  | 0.001 |  |
| FEV_1_ ≥ 80% | 63 | *Reference* |  |  | *Reference* |  |  |
| FEV_1_ < 80% | 55 | 2.04 (1.31-3.17) |  |  | 2.27 (1.40-3.68) |  |  |

Abbreviation: Combination therapy: immune checkpoint inhibitors combined with other anticancer therapy, included chemotherapy, anti-angiogenesis, or tyrosine kinase inhibitor; ECOG PS: Eastern Cooperative Oncology Group performance status; FEV_1_: forced expiratory volume in 1 second; FEV_1_ pred(%): percentage of predicted FEV_1_; FVC: forced vital capacity; HR: hazard ratio; ICI: immune checkpoint inhibitors; ICI-pneumonitis: immune checkpoint inhibitor related pneumonitis; PD-L1: programmed cell death ligand-1.

**Supplementary Figure 8: Kaplan–Meier curve of OS in patients received ICI without driver mutation**

**
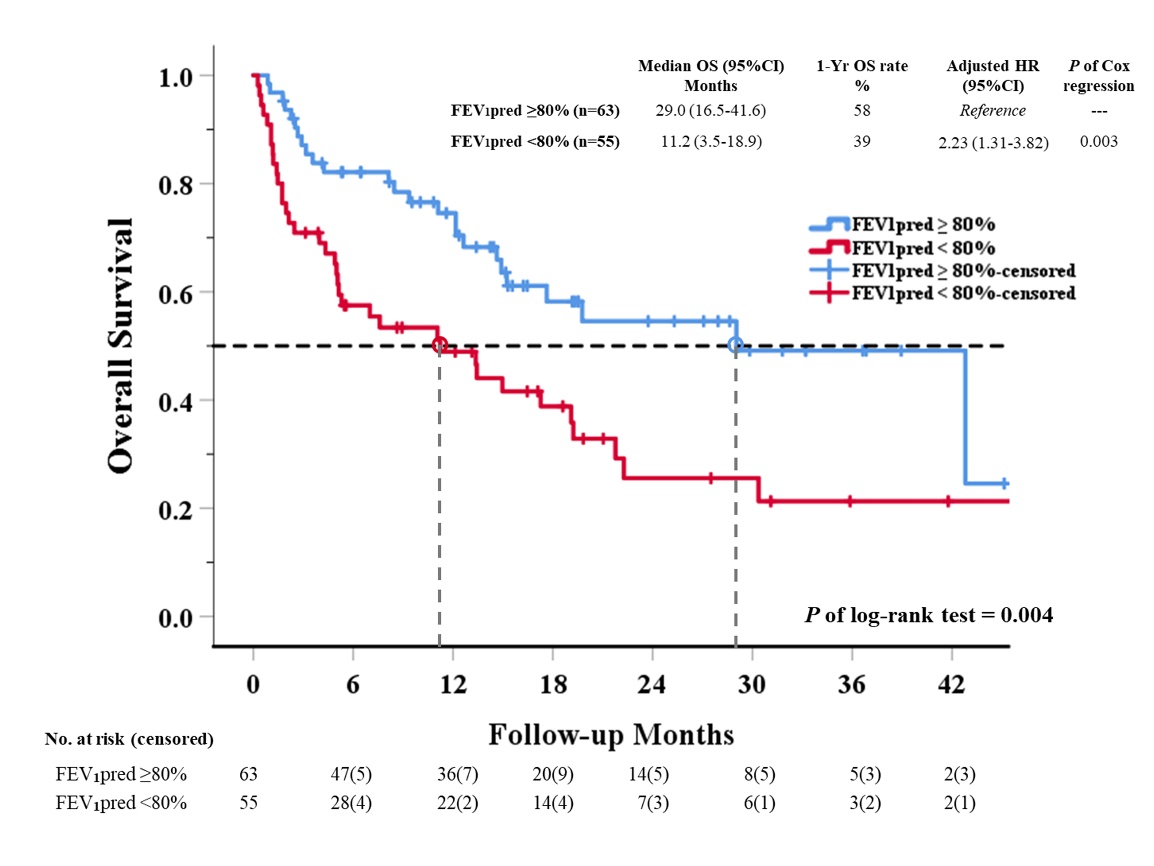
**

**Supplementary Table 12: Cox regression model of OS in patients received ICI without driver mutation**

|  |  | **Univariate analysis** | |  | **Multivariable analysis(*P*<0.1)** | |  |
| --- | --- | --- | --- | --- | --- | --- | --- |
| **Variable** | **N** | **HR (95% CI)** | ***P* value** |  | **HR (95% CI)** | ***P* value** |  |
| Age at ICIs treatment |  |  | 0.591 |  |  |  |  |
| <70 yrs | 83 | *Reference* |  |  |  |  |  |
| ≥70 yrs | 35 | 0.86 (0.48-1.52) |  |  |  |  |  |
| Gender |  |  | 0.801 |  |  |  |  |
| Female | 34 | *Reference* |  |  |  |  |  |
| Male | 84 | 1.07 (0.62-1.85) |  |  |  |  |  |
| Smoking history |  |  | 0.688 |  |  |  |  |
| No | 43 | *Reference* |  |  |  |  |  |
| Yes | 75 | 0.90 (0.54-1.50) |  |  |  |  |  |
| ECOG PS |  |  | 0.001 |  |  | 0.006 |  |
| 0 | 47 | *Reference* |  |  | *Reference* |  |  |
| 1-2 | 71 | 2.68 (1.51-4.77) |  |  | 2.40 (1.29-4.46) |  |  |
| Stage |  |  | 0.064 |  |  | 0.022 |  |
| IVA | 45 | 0.60 (0.35-1.03) |  |  | 0.50 (0.27-0.90) |  |  |
| IVB & IVC | 73 | *Reference* |  |  | *Reference* |  |  |
| Initial Brain metastasis |  |  | 0.244 |  |  |  |  |
| No | 91 | *Reference* |  |  |  |  |  |
| Yes | 27 | 1.41 (0.79-2.50) |  |  |  |  |  |
| Initial Lung metastasis |  |  | 0.546 |  |  |  |  |
| No | 82 | *Reference* |  |  |  |  |  |
| Yes | 36 | 1.18 (0.69-2.00) |  |  |  |  |  |
| Initial Liver metastasis |  |  | 0.017 |  |  | 0.017 |  |
| No | 105 | *Reference* |  |  | *Reference* |  |  |
| Yes | 13 | 2.39 (1.17-4.90) |  |  | 2.62 (1.19-5.75) |  |  |
| Pathology |  |  | 0.247 |  |  |  |  |
| Squamous cell carcinoma | 20 | *Reference* |  |  |  |  |  |
| Adenocarcinoma | 86 | 0.69 (0.37-1.29) |  |  |  |  |  |
| PD-L1 expression |  |  | 0.740 |  |  |  |  |
| <50% | 51 | *Reference* |  |  |  |  |  |
| ≥50% | 33 | 1.11 (0.58-2.14) |  |  |  |  |  |
| First-line therapy |  |  | 0.092 |  |  | 0.758 |  |
| No | 68 | *Reference* |  |  | *Reference* |  |  |
| Yes | 50 | 0.63 (0.37-1.08) |  |  | 0.91 (0.49-1.68) |  |  |
| Prior radiotherapy |  |  | 0.101 |  |  |  |  |
| No | 47 | *Reference* |  |  |  |  |  |
| Yes | 71 | 1.56 (0.92-2.65) |  |  |  |  |  |
| Prior lung surgery |  |  | 0.118 |  |  |  |  |
| No | 82 | *Reference* |  |  |  |  |  |
| Yes | 36 | 0.63 (0.36-1.12) |  |  |  |  |  |
| ICIs regimen |  |  | 0.008 |  |  | 0.136 |  |
| Monotherapy | 74 | 2.18 (1.23-3.86) |  |  | 1.66 (0.85-3.21) |  |  |
| Combination therapy | 44 | *Reference* |  |  | *Reference* |  |  |
| ICI-pneumonitis (all grade) |  |  | 0.004 |  |  | 0.006 |  |
| No | 113 | *Reference* |  |  | *Reference* |  |  |
| Yes | 5 | 4.53 (1.60-12.78) |  |  | 4.70 (1.55-14.22) |  |  |
| FEV_1_/FVC ratio |  |  | 0.748 |  |  |  |  |
| ≥0.7 | 118 | *Reference* |  |  |  |  |  |
| <0.7 | 33 | 1.10 (0.62-1.95) |  |  |  |  |  |
| FEV_1_ pred(%) |  |  | 0.005 |  |  | 0.003 |  |
| FEV_1_ ≥ 80% | 63 | *Reference* |  |  | *Reference* |  |  |
| FEV_1_ < 80% | 55 | 2.08 (1.25-3.47) |  |  | 2.23 (1.31-3.82) |  |  |

Abbreviation: Combination therapy: immune checkpoint inhibitors combined with other anticancer therapy, included chemotherapy, anti-angiogenesis, or tyrosine kinase inhibitor; ECOG PS: Eastern Cooperative Oncology Group performance status; FEV_1_: forced expiratory volume in 1 second; FEV_1_ pred(%): percentage of predicted FEV_1_; FVC: forced vital capacity; HR: hazard ratio; ICI: immune checkpoint inhibitors; ICI-P: immune checkpoint inhibitor related pneumonitis; PD-L1: programmed cell death ligand-1.
